# Identifying *OGN* as a Biomarker Covering Multiple Pathogenic Pathways for Diagnosing Heart Failure: From Machine Learning to Mechanism Interpretation

**DOI:** 10.3390/biom14020179

**Published:** 2024-02-02

**Authors:** Yihao Zhu, Bin Chen, Yao Zu

**Affiliations:** 1 International Research Center for Marine Biosciences, Ministry of Science and Technology, Shanghai Ocean University, Shanghai 201306, China; 2 Key Laboratory of Exploration and Utilization of Aquatic Genetic Resources, Ministry of Education, Shanghai Ocean University, Shanghai 201306, China; 3 Department of Cardiology, Shanghai Sixth People’s Hospital Affiliated to Shanghai Jiao Tong University School of Medicine (Lin-gang), Shanghai 201306, China; 4 Marine Biomedical Science and Technology Innovation Platform of Lin-gang Special Area, Shanghai 201306, China

**Keywords:** heart failure, diagnostic biomarker, pathogenic mechanism, bioinformatics

## Abstract

Background: The pathophysiologic heterogeneity of heart failure (HF) necessitates a more detailed identification of diagnostic biomarkers that can reflect its diverse pathogenic pathways. Methods: We conducted weighted gene and multiscale embedded gene co-expression network analysis on differentially expressed genes obtained from HF and non-HF specimens. We employed a machine learning integration framework and protein–protein interaction network to identify diagnostic biomarkers. Additionally, we integrated gene set variation analysis, gene set enrichment analysis (GSEA), and transcription factor (TF)-target analysis to unravel the biomarker-dominant pathways. Leveraging single-sample GSEA and molecular docking, we predicted immune cells and therapeutic drugs related to biomarkers. Quantitative polymerase chain reaction validated the expressions of biomarkers in the plasma of HF patients. A two-sample Mendelian randomization analysis was implemented to investigate the causal impact of biomarkers on HF. Results: We first identified *COL14A1*, *OGN*, *MFAP4*, and *SFRP4* as candidate biomarkers with robust diagnostic performance. We revealed that regulating biomarkers in HF pathogenesis involves TFs (*BNC2*, *MEOX2*) and pathways (cell adhesion molecules, chemokine signaling pathway, cytokine–cytokine receptor interaction, oxidative phosphorylation). Moreover, we observed the elevated infiltration of effector memory CD4+ T cells in HF, which was highly related to biomarkers and could impact immune pathways. Captopril, aldosterone antagonist, cyclopenthiazide, estradiol, tolazoline, and genistein were predicted as therapeutic drugs alleviating HF via interactions with biomarkers. In vitro study confirmed the up-regulation of *OGN* as a plasma biomarker of HF. Mendelian randomization analysis suggested that genetic predisposition toward higher plasma *OGN* promoted the risk of HF. Conclusions: We propose *OGN* as a diagnostic biomarker for HF, which may advance our understanding of the diagnosis and pathogenesis of HF.

## 1. Introduction

Heart failure (HF) is a heterogeneous and life-threatening clinical syndrome encompassing symptoms such as breathlessness, excessive fatigue, and swollen ankles [1]. HF has been considered a global pandemic estimated to affect more than 64 million individuals worldwide. Its prevalence has continuously increased over the past decades, posing a great threat to public health [2,3]. Traditionally, HF was defined as a pathophysiological condition in which cardiac structure or function abnormality results in increased intra-cardiac pressures or reduced cardiac output at rest or during activities [4]. However, the traditional definition only mentioned the non-specific symptoms or signs in clinical practice but did not include objective indicators to support HF diagnosis [4,5]. In 2021, a universal consensus on the definition of HF was proposed, namely a clinical syndrome with symptoms and/or signs caused by structural and/or functional cardiac abnormalities and corroborated by elevated natriuretic peptide levels and/or objective evidence of pulmonary or systemic congestion by diagnostic modalities [4], which was conceptually comprehensive and clinically practical with at least one objective detection indicator included. Notably, the elevated levels of natriuretic peptides, including the B-type natriuretic peptide (BNP) and N-terminal pro-B-type natriuretic peptide (NT-proBNP), were enrolled in this newly proposed definition of HF, which suggested the pivotal role of objective detective signs in HF recognition.

Biomarker-guided diagnosis and management have gained popularity in clinical application [5], and the natriuretic peptide has become a golden standard widely used in HF diagnosis or prognostic evaluation [6]. Nevertheless, the elevated concentrations of natriuretic peptides do not always meet the commands of diagnosing HF in clinics because it might be affected by numerous factors, such as renal failure, pulmonary embolism, and obesity [7]. Previously, evidence has shown that a deterioration in renal function significantly elevates the concentrations and associations of natriuretic peptides, indicating that the clinical interpretation of increased natriuretic peptides should consider renal function [8]. In the condition of acute pulmonary embolism, the concentrations of natriuretic peptides were also identified to increase as well as predict adverse prognostic outcomes [9]. Additionally, it has been demonstrated that obese patients with HF show a state of decreased levels of natriuretic peptides. Thus, lower thresholds of natriuretic peptide levels should be made to meet the diagnosis of obese individuals with HF [10]. Therefore, the application of natriuretic peptides remains limited in HF because of its vulnerability to other pathophysiologic conditions, indicating that exploring novel biomarkers that could provide additional diagnostic utility is urgently needed [11]. Given the complex pathophysiology of HF, which ranges from cardiac dysfunction to extra-cardiac alteration, emerging HF biomarkers that tackle different pathophysiological processes could significantly benefit the risk stratification and management of patients with HF [12]. In the past decades, two novel identified HF biomarkers, Galectin-3 (Gal-3) and tumorigenesis-2 (sST2), have yet to prove their utility in the clinics [13]. Moreover, growing evidence suggested that the multi-biomarker panel strategy, combining novel biomarkers with well-established ones, showed both strong diagnostic and prognostic values compared to a single-biomarker approach [5,14]. Hence, we hypothesized investigating newer specific diagnostic biomarkers together with elucidating their regulated pathogenic pathways involved in HF, which could provide candidates for the multi-biomarker strategy, thus deepening our understanding of molecular alterations in the progression of HF.

Given the explosion of biomedical big data, there is growing research enthusiasm in utilizing bioinformatics approaches for mining high-throughput RNA sequencing (RNA-seq) or single-cell RNA-seq (scRNA-seq) data, which make us better understand the altered molecular features in HF at tissue or cell levels [15]. With these rapidly developed sequencing techniques and computational analysis approaches, novel biomarkers and mechanisms could be rapidly identified rather than speckled through traditional hypothetical-driven research [5,15], bringing new insight into understanding the physiopathology of HF and developing the diagnosis or clinical management. Although several bioinformatics studies have already tackled exploring the underlying biomarkers and molecular mechanisms of HF [16,17,18,19], apparent limitations existed mainly due to the lack of comprehensive multi-datasets analysis from both tissue and single-cell views, rational speculation of the biomarkers-regulated pathways, and external experimental validation of biomarkers on testing patients’ samples. Considering these shortcomings in previous research, we intended to mine novel candidate biomarkers of HF as well as explicitly interpret their mechanisms (including upstream and downstream ways) that participated in HF via the in silico analysis on multiple RNA-seq datasets (including bulk RNA-seq and scRNA-seq) and in vitro validation on plasma collected from HF patients and control cases.

In this study, multiple transcriptional datasets derived from patients with HF and control, including four expression profiles (GSE141910, GSE57338, GSE42955, GSE135055) and one sc-RNA seq dataset (GSE121893), were acquired from the Gene Expression Omnibus (GEO) database. Training the cohort (GSE141910) for major analysis and test cohorts (GSE57338 and GSE42955) for validation were then processed and obtained, respectively. GSE135055 served as an external validation cohort. First, the differential expression genes (DEGs) between the HF and control were identified. Second, weighted gene co-expression network analysis (WGCNA) and multiscale embedded gene co-expression network analysis (MEGENA) complementing each other via different algorithms were performed on the DEG expression profiles to filter key gene modules. Subsequently, Disease Ontology (DO), Gene Ontology (GO), and Kyoto Encyclopedia of Genes and Genomes (KEGG) enrichment analyses were conducted on the overlapping genes between WGNCA-identified and MEGNA-identified modules, and genes significantly enriched in HF-related terms were retained. Fourth, the retained genes were subjected to a machine learning-based integration workflow and protein–protein interaction (PPI) network selection. Fifth, hub genes performed with robust diagnostic in the training and test cohorts were generated. Hub genes’ expression and correlation patterns were evaluated in the training and test cohorts. Sixth, sc-RNA seq analysis was conducted on GSE121893 to uncover the cell-specific expression patterns of hub genes. Seventh, together with gene set variation analysis (GSVA), gene set enrichment analysis (GSEA), transcription factor (TF) prediction, and single-sample gene set enrichment analysis (ssGSEA), we systemically identified the pathways co-regulated by hub genes involved in HF. Then, we used the ssGSEA approach to quantify the infiltration levels of immune cells in HF to screen crucial immune cell types highly correlated to the hub genes. Eighth, the therapeutic drugs targeting hub genes and TFs were verified using molecular docking. Finally, quantitative reverse transcription PCR (RT-qPCR) was utilized to explore the relative mRNA expression levels of hub genes in plasma collected from patients with HF, thereby developing plasma biomarkers with HF. Based on large-scale genome-wide association studies (GWASs), a two-sample Mendelian randomization (MR) analysis was further performed to investigate the causal effect of hub genes on the risk of HF. The overall study flow chart is shown in Figure S1.

## 2. Materials and Methods

### 2.1. Gathering and Processing of Gene Expression Profiling Datasets

Three gene expression profiles (GSE141910, GSE57338, GSE42955, GSE135055) of left ventricles derived from HF patients were retrieved from the Gene Expression Omnibus (GEO) database (https://www.ncbi.nlm.nih.gov/geo/, accessed on 30 November 2022). GSE141910 was the training group in this study, which included 200 HF and 166 non-HF (control) samples [20]. We removed the batch effect of GSE57338 and GSE42955 and merged them into the test group using the “combat” algorithm of the R packages “sva” (version 3.44.0), containing 201 HF and 141 non-HF (control) samples [21,22]. The removal of batch effect on GSE57338 and GSE42955 was evaluated via the principal component analysis (PCA) plot, as shown in Figure S2. Furthermore, we selected GSE135055 (21 HF and 9 control samples) as an external validation group [23]. We then performed log transformation on the gene expression profiles and utilized the function “normalizeBetweenArrays” of the R package “limma” (version 3.52.2) to execute data normalization [24]. The detailed information on these enrolled datasets is summarized in Table 1. Moreover, the clinical characteristics included in the datasets can be found in Tables S1–S4.

### 2.2. Partial Least Squares Discriminant Analysis, DEGs Identification, and KEGG Pathway Enrichment Analysis

To evaluate the separation between control and HF groups, partial least squares discriminant analysis (PLS-DA) was performed using the R package “mixOmics” (version 6.20.0) [25]. The DEGs between the HF group and the control group (HF versus control) were identified by the R package “limma” [24]. The cut-off criteria of DEGs were set as |log2Fold Change (log2FC)| > 1 and adjusted *p*-value < 0.05 [26]. Kyoto Encyclopedia of Genes and Genomes (KEGG) pathway enrichment analysis was then conducted on DEGs through the R package “clusterProfiler” (version 4.7.1) [27]. The enrichment KEGG pathway showed with *q* value < 0.05 was considered statistically significant.

### 2.3. Weighted Gene Co-Expression Network Analysis on DEGs

The R package “WGCNA” (version 1.71) was utilized to construct a weighted gene co-expression network on DEGs, which was suitable for further screening functional gene modules highly associated with HF [28]. The soft threshold power (β = 1~20) was first determined to establish a scale-free topology. The weighted adjacency matrix was then generated and transformed into a topological overlap matrix (TOM). Moreover, the dissTOM was obtained for hierarchical clustering, and the dynamic tree-cutting method was adopted to screen various modules clustered on gene similarity. Here, the parameters “minModuleSize” and “MEDissThres” were set to 60 and 0.3, respectively. Subsequently, we related the recognized modules to two traits (control and HF). Genes in the module that displayed high relevance with HF were selected for further analysis.

### 2.4. Multiscale Embedded Gene Co-Expression Network Analysis on DEGs

The R package “MEGENA” (version 1.3.7) was performed to identify gene co-expression networks on DEGs by Planar Filtered Networks (PFNs) construction, thus dissecting into multiscale gene modular structures in HF [29]. The input parameters were set to default. First, we obtained a fast PFN via calculating, filtering, and clustering correlations between each gene pair based on the DEG expression profiles of HF. Multiscale clustering analysis (MCA) was then conducted on PFN, and sub-modules were identified from the connected components of the initial PFN as the parent modules. Next, we implemented multiscale hub analysis to detect key hubs of individual modules. The identified modules with large gene sizes were retained for later selection.

### 2.5. DO, GO, and KEGG Enrichment Analyses on Crucial Gene Modules

Taking the intersection between the significantly co-expressed gene modules identified by WCGNA and MEGENA, we obtained overlapping genes for functional and pathway annotation. Annotation of human diseases, biological functions, and signaling pathways could reflect whether the overlapping genes in these identified modules are directly enriched in HF-related terms [30], contributing to our understanding of these genes’ role in HF. Accordingly, we systemically performed Human Disease Ontology (DO), Gene Ontology (GO), and KEGG enrichment analyses on these overlapping genes using the R packages “clusterProfiler” and “DOSE” (version 3.24.2) [27,31]. A *q* value < 0.05 was considered the cut-off criterion for identifying significant enrichment terms.

### 2.6. Feature Selection via Machine Learning-Based Integration

The overlapping genes co-identified via WGCNA and MEGENA were subjected to a machine learning-based integration pipeline [32]. A total of 12 machine learning models for integration included least absolute shrinkage and selection operator (Lasso), Ridge, elastic network (Enet), stepwise multiple generalized linear model (Stepglm), support vector machine (SVM), generalized linear model by likelihood-based boosting (glmBoost), linear discriminant analysis (LDA), partial least squares regression for generalized linear models (plsRglm), Random Forest (RF), gradient boosting machine (GBM), eXtreme Gradient Boosting (XGBoost), and naïve Bayes. Herein, the machine learning-based integration pipeline applied one algorithm for feature selection and another for constructing an integrative classification prediction model under the 10-fold cross-validation (CV), generating 113 algorithm combinations in this study. The execution of the machine learning-based integration pipeline involves the following procedures. (1) We first performed Z-score transformation on the expression profiles of training and testing cohorts, which could enhance comparability between diverse cohorts and accelerate running speed. (2) In our integrative ML-based framework, four algorithms with feature selection capability (Lasso, RF, Stepglm, glmBoost) were initially conducted to narrow down the genes. Then, we adopted eight other algorithms (Ridge, Enet, SVM, LDA, plsRglm, GBM, XGBoost, NaiveBayes) to fit prediction models based on the genes selected via the four algorithms, respectively. In total, 113 model combinations were used to tune hyperparameters and fit models under a 10-fold CV framework. (3) All model combinations were subsequently tested in training and testing cohorts. For the performance evaluation of each model, the AUC score across these cohorts was computed. The model with the highest average AUC within the training and testing cohorts was regarded as optimal. Feature genes were subsequently obtained via the best-performing model.

### 2.7. Construction of Protein-Protein Interaction Network

Leveraging the feature genes generated from machine learning-based integration, a protein–protein interaction (PPI) network was constructed using the Search Tool of the Retrieval of Interacting Genes (STRING) database and then visualized by Cytoscape software (version 3.9.1) [33,34]. We further screened out the top 20 genes with higher connectives in the PPI network using five ranking algorithms of Cytoscape plug-in cytoHubba, respectively. The Degree, Maximum Neighborhood Component (MNC), Maximal Clique Centrality (MCC), Edge Percolated Component (EPC), and Density of Maximum Neighborhood Component (DMNC) algorithms were used to screen genes in the PPI network [35]. We noted the intersected genes co-selected in five algorithms as pivotal genes of HF for further selection and assessment.

### 2.8. Diagnostic Abilities, Expression Levels, and Correlation Pattern of Hub Genes

To investigate the diagnostic performances of pivotal genes in HF, we calculated the AUC score of receiver operating characteristic curve (ROC) analysis using the R package “pROC” (version 1.18.0) to measure the classification accuracy of the HF group and control group [36]. Genes performed with an AUC higher than 0.85 distinguishing HF from control in both the training and test cohorts were considered hub genes, potentially serving as biomarkers of HF diagnosis. We then utilized the R package “glmnet” (version 4.1-4) to construct logistic regression models to evaluate the joint diagnostic abilities of hub genes in the training and test cohorts. To compare the diagnostic abilities of our identified hub genes with clinical biomarkers, we retrieved a panel of current HF biomarkers, including myocardial stress/injury-related (BNP, NT-proBNP, TnT, TnI), neurohormone-related (MR-proADM, AVP, Peptide), myocardial remodeling-related (sST2, Gal-3, MMP, GDF-15), inflammatory-related (CRP, IL-6, TNF-α, PCT), and renal function injury-related (Cys C, NGAL) biomarkers. Comparison of the predictive power of hub genes and current biomarkers was then assessed via AUC score within the training and testing cohorts. Using the R package “ggpubr” (version 0.4.0), the significant over-expression pattern of hub genes in HF compared to the control was shown in the violin plot and verified in the test cohort. Correlation analyses between hub genes in the training and test cohorts were performed and visualized using the R package “PerformanceAnalytics” (version 2.0.4).

### 2.9. Single-Cell RNA-seq Data Processing

To explore the specific cell expression patterns of hub genes in HF, we obtained one scRNA-seq dataset (GSE121893) derived from six patients with HF [37], including 1682 cardiomyocytes (CMs) and 2539 non-cardiomyocytes (NCMs). The R package “Seurat” (version 4.1.1) was implemented to process and analyze single-cell RNA-seq datasets [38]. First, we conducted quality control and normalization of the data. Cells with unique feature counts less than 200 and mitochondrial proportions larger than 5% were eliminated in further analysis. The “Log normalize” function was then employed to normalize the filtered data. Second, we obtained the top 2000 highly variable genes identified by the function “FindVariableFeatures” and scaled the data as the pre-processing procedure of subsequent dimensional reduction. Third, we performed principal component analysis (PCA) on the scaled data with the top 2000 highly variable genes as input. Combining the JackStraw and Elbow plots, we determined the optimal components (1 to 15) to cluster cells. Fourth, the Uniform Manifold Approximation and Projection (UMAP) algorithm was executed to display the cell clusters. We then assigned five cell types (CM, cardiomyocyte; EC, endothelial cell; FB, fibroblast; SMC, smooth muscle cell; MP, macrophage) to the identified clusters according to the expression distributions of gene markers. The markers of five cell populations were listed: CM (*TNNT2*, *MYH7*, *MYOM1*, *ACTN2*), EC (*VWF*, *ENG*, *RGCC*, *EMCN*), FB (*FN1*, *VIM*, *DCN*, *VCAN*), SMC (*ACTA2*, *MYLK*, *MYL6*, *MYL9*), and MP (*CCR2* negative, *CD74*, *ITGAM*, *MRC1*) [37]. We also used the R package “AUCell” (version 1.22.0) to calculate the AUCell scores of the specific gene markers for each cell to reflect the marker activities within the five cell populations [39]. The R package “Nebulosa” (version 1.6.0), a novel gene expression signal recovering approach on weighted kernel density estimation, was utilized to uncover the expression distributions of hub genes in the five annotated cell populations [40].

### 2.10. GSVA and GSEA of Hub Genes

Herein, we integrated gene set variation analysis (GSVA) and gene set enrichment analysis (GSEA) to investigate the underlying pathways of each hub gene in HF. The reference gene sets of GSEVA and GSEA were the KEGG subset of canonical pathways collected from the Human Molecular Signatures Database (MSigDB) (http://www.gsea-msigdb.org/gsea/msigdb/collections.jsp, accessed on 30 November 2022) [41]. We first performed GSVA on the expression profiles of HF leveraging the R package “GSVA” (version 1.44.2) [42]. Then, the R package “limma” was conducted to compare the GSVA scores of pathways between the low-expression and high-expression groups according to the medium value of the hub gene. *p* value < 0.05 and |t| > 2 were considered as cut-off criteria for identifying the significant activated (t > 2) or suppressed (t < 2) pathways [43]. Meanwhile, GSEA was conducted for each hub gene based on the expression profiles of HF using the R package “clusterProfiler” [27,43]. The GSEA pathways with |normalized enrichment score (NES)| > 1, *p* value < 0.05, and false discovery rate (FDR) < 0.25 were regarded as statistically significant enrichment [44]. NES larger than 1 and lower than 1 indicated the activation and suppression of the enriched pathway, respectively. The statistically significant overlap between the GSEA and GSVA on each hub gene was identified as the common pathways (activated or suppressed) co-regulated by hub genes in HF. Moreover, the R package “CBNplot” (version 0.99.2) was employed to construct and visualize the gene regulatory network (GRN) via Bayesian network (BN) inference from expression profiles and enrichment analysis [45].

### 2.11. Expression Levels of Predicted TFs and Their Interaction with Hub Gene-Related Pathways

To gain insight into the upstream mechanism regulating hub genes, we acquired the TRRUST v2 database (www.grnpedia.org/trrust, accessed on 23 March 2023) to search for underlying transcription factors (TFs) interacting with hub genes [46]. The top 10 predicted TFs with higher-ranked sentences in the database were retained. TFs that were up-regulated in HF may actively target hub genes. Next, we performed a correlation analysis between the key TFs and hub genes. Additionally, single-sample gene set enrichment analysis (ssGSEA) was employed using the R packages “GSVA” and “GSEABase” (version 1.58.0) to quantify the enrichment levels of the pathways related to hub genes in HF samples [42,47]. Subsequently, we conducted a correlation analysis between the key TF expressions and pathway enrichment levels.

### 2.12. Evaluation of Immune Cell Characteristics in HF via ssGSEA and Identifying Immune Cell Types Highly Correlated with Hub Genes

To explore whether the immune landscapes were altered in HF, we employed the ssGSEA algorithm to quantify the infiltration levels of 28 immune cell types based on gene expression profiles [48,49]. Using ssGSEA by interrogating the expression levels of reported specific genes of adaptive and innate immune cell types, we computed the infiltration levels of 28 immune cell types per sample. Next, we integrated Lasso and RF algorithms to obtain key immune cell types from 28 immune cell types [50]. Lasso and RF were constructed under 10-fold cross-validation, and the setting parameters of RF were 800 decision trees. Using the R package “glmnet”, a Lasso regression model was established under the optimal penalization coefficient (λ), and immune cell types with regression coefficients larger than zero were retained [51]. The R package “randomForest” (version 4.6-7) was utilized to construct an RF classifier using 586 decision trees that was performed with a minimum classification error rate, and immune cell types with mean decrease Gini scores larger than five were screened [52]. Taking the intersection between Lasso and RF, we identified a final set of key immune cell types in HF. Subsequently, we assessed the correlation patterns between hub genes and key immune cell types that were significantly up-regulated in HF compared to control.

### 2.13. Molecular Docking of Predictive Drugs Targeting Hub Genes and TFs

First, candidate small-molecule agents with targets (hub genes or key TFs) were predicted using the Drug Signatures Database (DSigDB) [53]. Molecular docking, a computational approach for exploring the interaction between receptors and ligands, was executed to evaluate the binding affinities of the selected candidate drugs to their targets [54]. AutoDock Vina (version 1.1.2) was used to perform molecular docking analysis [55]. Second, the 2D structures of molecule ligands (candidate drugs) were downloaded from the PubChem database (https://pubchem.ncbi.nlm.nih.gov/, accessed on 26 March 2023) in SDF formats. The ChemBio3D was performed to minimize energy on molecule ligands and export their 3D structures. AutoDock Vina then converted the molecule ligands (mol2 format) into PDBQT format for further docking analysis. Third, the 3D structures of protein receptors (hub genes and key TFs) were passed through the Protein Data Bank (PDB) (https://www.rcsb.org/, accessed on 26 March 2023). We used the PyMOL (version 2.4.0) to remove water molecules and ligands from proteins. The protein receptors (PDB format) were imported into the AutoDock Vina and exported in the PDBQT format after polar hydrogenation and docking site setting. Herein, the parameters of the docking site, including X-Y-Z coordinates and grid size, were adjusted to include the active pocket that completely binds molecule ligands. Finally, AutoDock Vina was executed to dock the molecule ligand and protein receptors 20 times. Confident docking results with the lowest binding affinity and Root Mean Squared Error (RMSE) lower than 2 Å were retained and subsequently visualized by PyMOL.

### 2.14. Quantitative Reverse Transcription PCR

To investigate whether the hub genes could serve as plasma biomarkers of HF, we collected the whole blood specimens stored in ethylenediaminetetraacetic acid (EDTA) tubes, derived from three HF patients and three non-HF controls in the Shanghai Sixth People’s Hospital Affiliated to the Shanghai Jiao Tong University School of Medicine (Lin-gang). The detailed information of patients, including gender, age, disease diagnosis, HF grade, HF type, ejection fraction (EF), and NT-proBNP concertation, are shown in Table S5. The types of HF of the three patients include congestive heart failure and ischemic heart failure. Whole blood was first centrifuged at 3500 relative centrifugal force (RCF) for 10 min at room temperature, and plasma was separated. RNA was extracted from the collected plasma using a BIOG cfRNA Easy Kit (Changzhou Baidai, Changzhou, China). Subsequently, we utilized the PrimeScript RT Reagent Kit with a gDNA Eraser Kit (Takara, Kusatsu, Japan) to reverse transcribe RNA into cDNA. Quantitative reverse transcription PCR (qRT-PCR) analysis was performed on cDNA using the AceQ Universal SYBR qPCR Master Mix (Vazyme, Nanjing, China). The relative expression of hub genes was calculated by the 2-DDCt method adjusted to β-actin. The primer sequences of four hub genes and β-actin are shown in Table S6.

### 2.15. Two-Sample Mendel Randomization Analysis

We sought to explore the causal effect of plasma *OGN* (exposure) on HF (outcome) using a two-sample Mendel Randomization (MR) analysis. Herein, we used the R package “TwoSampleMR” (version 0.5.7) to analyze. The exposure data (plasma *OGN*; ID: PRJEB15197) from 35,559 Icelanders and outcome data (HF, ID: ebi-a-GCST009541) from 47,309 Europeans with HF reported were retrieved from the deCODE genetics (https://www.decode.com/summarydata/, accessed on 27 December 2023) and the IEU Open GWAS data source (https://gwas.mrcieu.ac.uk/, accessed on 27 December 2023), respectively. Single-Nucleotide Polymorphisms (SNPs) used as instrumental variables (IVs) in MR analysis should be subject to three assumptions [56]: (1) SNPs are closely related to exposure; (2) SNPs are independent of cofounders of exposure and outcome; (3) SNPs only affect the outcome through exposure. Accordingly, the function “extract_instruments” was utilized to retain the SNPs that were significantly associated with *OGN* (*p*-value < 5 × 10^−6^) but not with HF (*p*-value > 0.05) [57]. SNPs with linkage disequilibrium (LD) R^2^ larger than 0.01 within a cropping range of 5000 Kb were excluded [57]. Next, the function “harmonise_data” was leveraged to harmonize the exposure and outcome data. Two-sample MR analysis was subsequently performed via the function “mr”. Five default methods were adopted: MR Egger, Weighted median, Inverse variance weighted (IVW), Simple mode, and Weighted mode. The R package “forestploter” (version 1.1.1) [58] was employed to generate a forest plot showing the Wald ratio-estimated odds ratio (OR) of *OGN* on HF. The IVW approach, with the highest statistical power [56,57], was selected to illustrate the association between the level of plasma *OGN* and the risk of AMI. We employed heterogeneity, horizontal pleiotropy, and leave-one-out tests as sensitivity analysis as well as serving as a reliability evaluation of MR analysis.

### 2.16. Statistical Analysis

Statistical analysis was performed using R programming (version 4.2.2). A Wilcoxon rank-sum test was used to analyze the two groups’ differential expression levels of hub genes. We performed Pearson’s correlation analysis on expression levels of hub genes. Spearman’s correlation analysis was conducted to explore the relationship between infiltrations of immune cell types and expressions of hub genes. Spearman’s correlation analysis was also utilized to assess the interaction between expressions of TFs and ssGSEA scores of pathways. The results of qRT-PCR were presented as mean ± standard error (SD), and an unpaired t-test was carried out to compare the mean expressions of hub genes between HF and control. *p* value < 0.05 was considered statistically significant.

## 3. Results

### 3.1. DEGs Mainly Participated in the Extracellular Matrix and Immune-Related Pathways in HF

**Figure 1:**
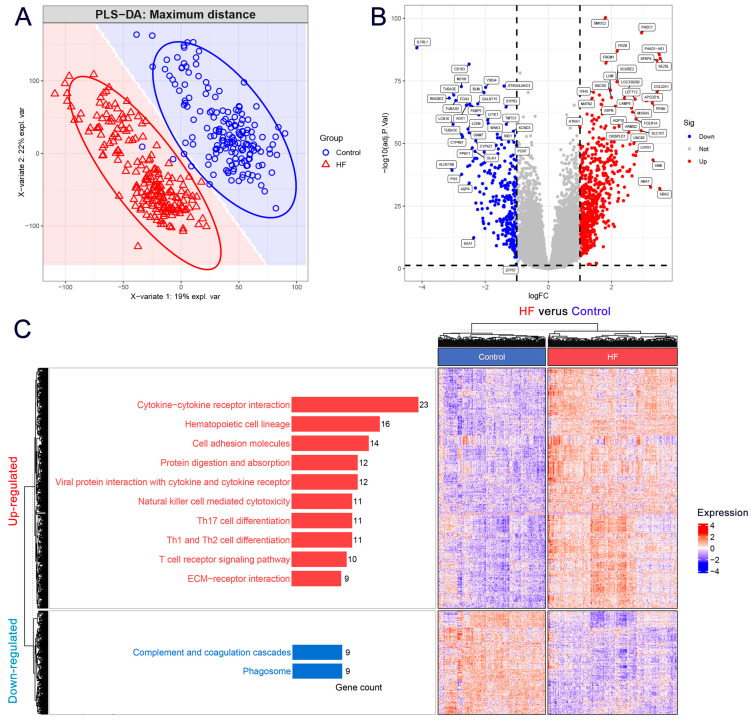
Identification of DEGs of HF from the training cohort with *n* = 366 (200 HF and 166 non-HF controls) and KEGG enrichment analysis. (**A**) The plot of PLS-DA scores using two components shows the distinct separation between the HF and control groups. (**B**) The Volcano plot of DEGs with a significant change in |log2FC| > 1 and FDR < 0.05. The red and blue dots represent up-regulated and down-regulated DEGs, respectively. (**C**) The clustering heatmap of DEGs among the HF group and control group. Left panel annotation: The significant KEGG enrichment pathways of up- and down-regulated DEGs (all *q* < 0.05).

To investigate the differences between HF and control groups, we performed PLS-DA on the expression profile of the training cohort. Figure 1A shows a distinct sample separation between HF and control groups, which could be used for DEG analysis. We then identified 926 DEGs (HF group versus control group) with log2FC larger than 1 and adjusted *p* value lower than 0.05, including 648 up-regulated DEGs and 278 down-regulated DEGs (Figure 1B). Detailed information on these 926 DEGs can be found in Table S7. KEGG enrichment analysis was then performed to understand better the potential functions of these selected DEGs. The significantly enriched pathways of up-regulated and down-regulated DEGs are shown in Figure 1C (left panel), respectively. The up-regulated DEGs were mainly involved in cytokine–cytokine receptor interaction, cell adhesion molecules, the T cell receptor signaling pathway, and ECM–receptor interaction. Interestingly, these enrichment pathways were largely related to the extracellular matrix (ECM) or immune-related functions, suggesting their crucial roles positively participated in HF. The down-regulated DEGs mainly participated in complement and coagulation cascades and the phagosome, which indicated that coagulation functions were abnormally altered in HF. We also observed the distinct expression patterns of DEGs in the clustering heatmap in Figure 1C (right panel), which illustrates that the DEGs could clearly distinguish the samples as HF from the control.

### 3.2. Two Crucial Modules Strongly Related to HF Were Identified by WGCNA on DEGs

**Figure 2:**
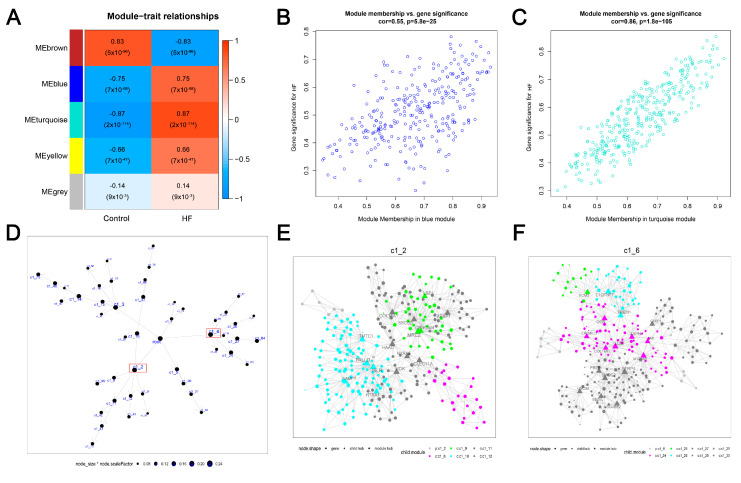
Crucial modules identified WGCNA and MEGENA from the training cohort with *n* = 366 (200 HF and 166 non-HF controls). (**A**) The heatmap showed the relationship between five WGCNA-identified modules and two traits (HF and control). Each cell includes the correlation coefficient and *p* value. (**B**) Scatterplots of high correlations between GS versus MM for HF in blue module from WGCNA. (**C**) Scatterplots of high correlations between GS versus MM for HF in turquoise modules from WGCNA. (**D**) The gene co-expression network of HF via MEGENA. Node stands for distinct modules, and the sizes of node and label are proportional to gene numbers. The two modules with the largest genes in the network are shown: c1_2 and c1_6 (highlighted in red boxes). (**E**) Intergenic connectivity of genes in c1_2 module. (**F**) Intergenic connectivity of genes in c1_6 module.

To understand the gene expression network in HF, we performed WGCNA to establish a weighted gene co-expression network based on the expression profiles of the identified 926 DEGs. First, a similarity matrix was generated by calculating gene pairwise correlation. We then evaluated soft threshold power (β) between 1 and 20 to construct a scale-free network. Second, the similarity matrix was converted into an adjacent matrix via the optimal β achieving 2 (Figure S3A). Furthermore, a negative correlation (R^2^ = 0.83, slope = 1.6) between log10(k) and log10 (p(k)) was observed in Figure S3B, indicating that the transformed adjacent matrix was close to a scale-free network for further analysis. Third, the topological overlap matrix (TOM) was generated using the adjacent matrix for dynamic tree-clustering on genes (Figure S3C), thus identifying gene modules with HF. Genes recognized in each WGCNA-identified module can be found in Table S8. The correlation between identified gene modules and two traits (HF and control) was calculated. As shown in Figure 2A, the ME turquoise and ME blue modules showed significantly positive correlations to HF (R > 0.7, *p* < 0.05). We also observed the significantly distinct distribution of gene significance (GS) across modules (*p* < 0.05, Figure S3D). Fourth, we performed intramodular analysis and found significantly positive correlations between module membership (MM) and gene significance (GS) in the ME turquoise (R = 0.55, *p* < 0.05) (Figure 2B) and ME blue modules (R = 0.86, *p* < 0.05) (Figure 2C). Finally, we considered the genes in the ME turquoise module (*n* = 356) and ME blue module (*n* = 298) as HF-related genes for later analysis.

### 3.3. Two Largest Crucial Modules of HF Were Identified by MEGENA on DEGs

To gain insight into a more biologically meaningful gene expression network in HF, we implemented MEGENA on the expression profiles of the 926 DEGs to establish a multiscale gene co-expression network. We first calculated and filtered the correlations between each gene pairwise of 926 DEGs. Subsequently, a fast PFN was calculated to filter and retain the significant correlation pairs. We then performed MCA on splitting PFN to identify sub-modules (summarized in Table S9). As shown in Figure 2D, 48 significant gene modules were identified (module significance *p* < 0.05), of which the c1_2 was the largest module of 288 genes, which was followed by the c1_6 module containing 276 genes. Moreover, we displayed the subnetworks of the c1_2 and c1_6 modules. As shown in Figure 2E,F, the c1_2 and c1_6 modules comprised six and eight child modules, respectively.

### 3.4. A Large Set of Overlaps between Crucial Modules Showed Close Associations with HF-Related Biological Functions and Pathways

**Figure 3:**
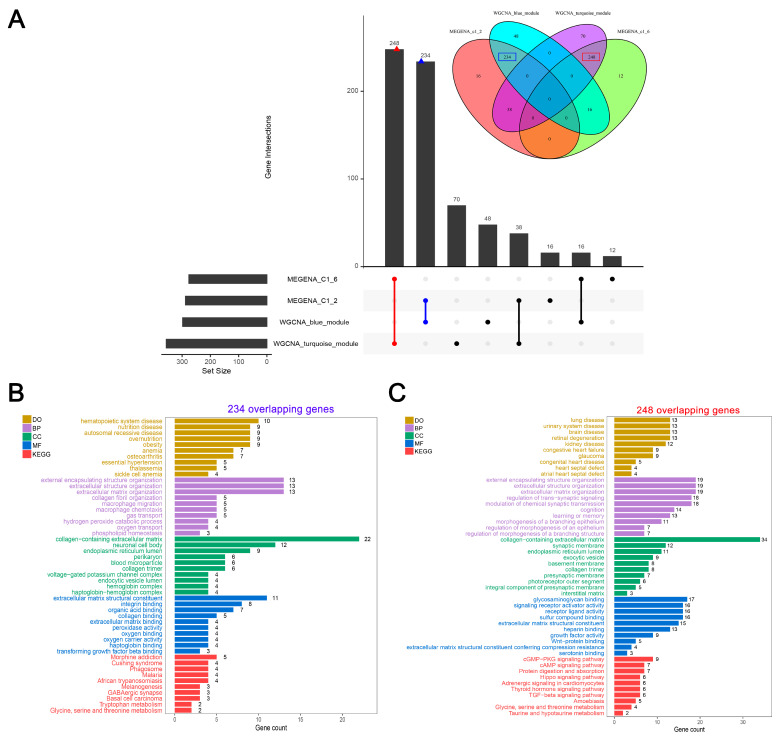
Overlap between crucial modules of WGCNA and MEGENA from the training cohort with *n* = 366 (200 HF and 166 non-HF controls). (**A**) The Upset plot and Venn plot show two large sets of overlapping (indicated by red and blue triangles) between 4 selected modules, including 2 crucial modules of WGCNA and 2 crucial modules of MEGENA. (**B**) DO, GO, and KEGG enrichment analysis of shared genes between the blue module and c1_2 module. (**C**) DO, GO, and KEGG enrichment analysis of shared genes between the turquoise module and c1_6 module.

Comparing the two significantly positive gene modules of WCGNA with the two significantly large gene modules of MEGENA, we obtained two large sets of intersections between these identified modules. As shown in Figure 3A, 248 genes were overlapping between the ME turquoise of WGCNA and the c1_6 module of MEGENA. We also observed that 234 genes intersected between ME blue of WGCNA and the c1_2 module of MEGENA. Enrichment analysis contributes to understanding whether the preservation of these modules between WGNCA and MEGENA could comprehensively represent the biological significance of HF. Subsequently, we conducted DO, GO, and KEGG enrichment analyses on the two sets of overlapping genes co-identified by WCGNA and MEGENA. DO enrichment analysis showed that the 234 genes largely participated in hematopoietic system disease, nutrition disease, autosomal recessive disease, overnutrition, and obesity (Figure 3B). GO enrichment analyses demonstrated that the 234 genes were highly associated with ECM, such as extracellular matrix structural constituent, collagen-containing extracellular matrix, and extracellular structure organization (Figure 3B). Furthermore, KEGG enrichment analysis revealed that 234 genes primarily participated in morphine addiction, Cushing syndrome, and phagosome (Figure 3B). DO enrichment analysis suggested that 248 genes were enriched in cardiac abnormality-related disorders, such as congestive heart failure, congenital heart disease, heart septal defect, and atrial heart septal defect (Figure 3C). It was also shown that 248 genes were mainly related to ECM-related functions, such as extracellular matrix structural constituent, collagen-containing extracellular matrix, and extracellular matrix organization. Notably, several pathways have been proven significant to HF progression, such as the cGMP-PKG signaling pathway [59], the cAMP signaling pathway [60], the Hippo signaling pathway [61], adrenergic signaling in cardiomyocytes [62], and the TGF-beta signaling pathway [63], were observed in the KEGG enrichment of 248 genes (Figure 3C). Taken together, we noticed that compared with 234 genes, 248 genes were enriched in more biological functions or pathways associated with HF, which was used as a gene set for further feature selection.

### 3.5. Machine Learning-Based Integration and PPI Network Analysis Screened 10 Pivotal Genes of HF

**Figure 4:**
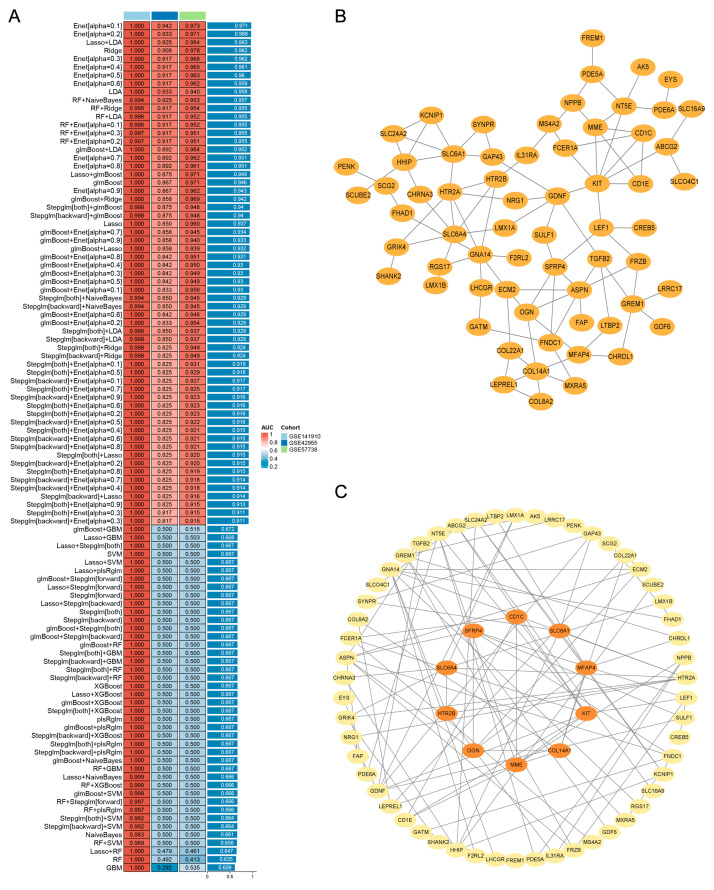
Pivotal genes were generated from the machine learning-based integration and PPI network. (**A**) A total of 113 machine learning-based prediction models performed feature selection on 210 genes in the training and test cohorts, and the AUC score of each model was then calculated. (**B**) PPI network of 134 feature genes. (**C**) The pivotal genes in the PPI network: Orange indicates 10 pivotal genes.

The expression profiles of 248 genes were subjected to the machine learning-based integration pipeline to filter pivotal genes of HF. Herein, we developed 113 predictive classification models fitted on the training and test cohorts via the CV framework. As shown in Figure 4A, the Enet (alpha = 0.1) with the highest mean AUC scores (0.971) performed within the training and test cohorts was considered the optimal classification model. Therefore, we obtained a set of 134 feature genes via the Enet (alpha = 0.1). To investigate the gene functional association, we subsequently constructed a 134 genes-dominant PPI network (Figure 4B). The genes showed interactions were retained in the network, and non-interactive genes were eliminated. Using the cytoHubba, we carried out the Degree, MNC, MCC, EPC, and DMNC algorithms to identify the network’s top 20 genes with higher connectives (Figure S4). Finally, we obtained an interactive genes-dominant PPI network and identified 10 pivotal genes (*KIT*, *SLC6A1*, *SLC6A4*, *COL14A1*, *MME*, *OGN*, *SFRP4*, *CD1C*, *MFAP4*, *HTR2B*) overlapping between the five algorithms (Figure 4C).

### 3.6. COL14A1, OGN, MFAP4, and SFRP4 Were Hub Genes as Candidate Diagnostic Biomarkers of HF

**Figure 5:**
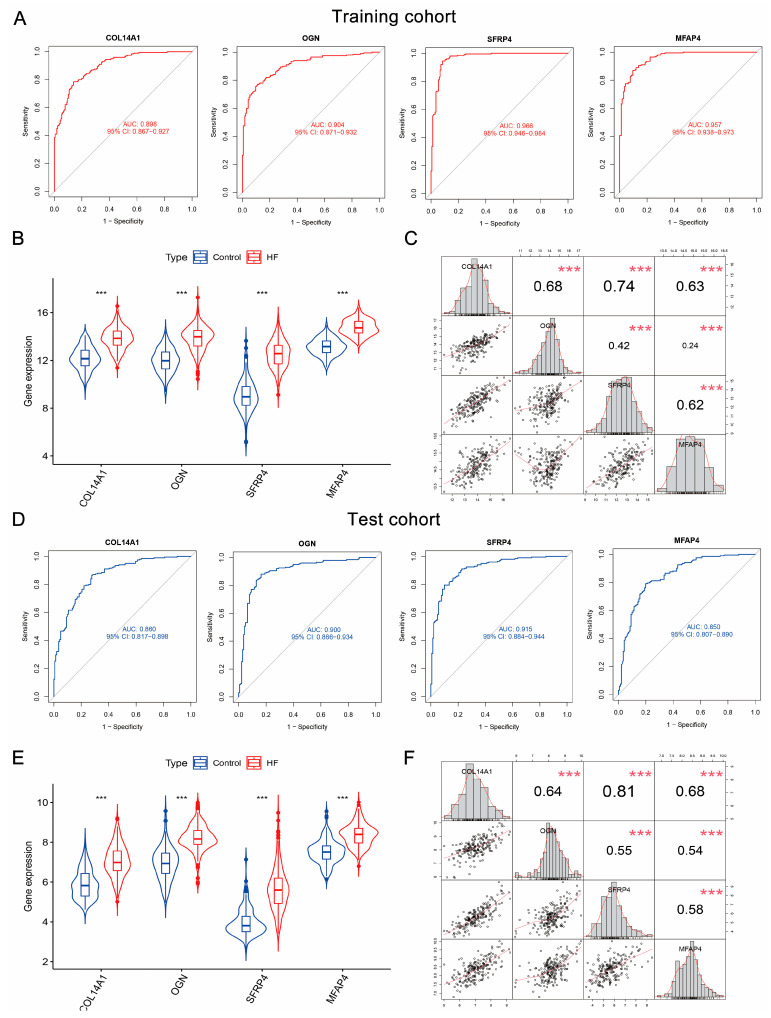
ROC curve analysis, expression levels, and correlations of four hub genes. (**A**) ROC curves of four hub genes in the training cohort. (**B**) Expression levels of four hub genes in HF compared to the control group in the training cohort. (**C**) Correlation heatmap of four hub genes expressed in HF in the training cohort. Red line indicates the fitted curve. (**D**) ROC curves of four hub genes in the test cohort. (**E**) Expression levels of four hub genes in HF compared to the control group in the test cohort. (**F**) Correlation heatmap of four hub genes expressed in HF in the test cohort. Red line (Figure 5C,F) indicates the fitted curve. ***, *p* < 0.001.

To assess the diagnostic abilities of 10 pivotal genes, we conducted ROC curve analysis in the training and test cohorts. The results demonstrated that *COL14A1*, *OGN*, *MFAP4*, and *SFRP4* achieved AUC scores all larger than 0.85 in the training cohort (Figure 5A and Figure S5A) and test cohort (Figure 5D and Figure S5B), suggesting the high sensitivity and specificity that distinguish HF from control. Accordingly, *COL14A1*, *OGN*, *MFAP4*, and *SFRP4* were considered hub genes of HF that could serve as underlying diagnostic biomarkers of HF. Additionally, combining four hub genes largely elevates the diagnostic performances within the training cohort (AUC = 0.988, Figure S5C) and test cohort (AUC = 0.935, Figure S5D). The diagnostic accuracy, correlations, and expression patterns of *COL14A1*, *OGN*, *MFAP4*, and *SFRP4* were further assessed within two datasets (GSE57338 and GSE42955) from the test cohort (Figure S6). Despite the different sample sources from the two datasets, robust diagnostic powers and elevated expressions of these genes were again verified, particularly *OGN* (all AUC > 0.9, Figure S6C,G). We subsequently compared the diagnostic performance of these four hub genes with current HF biomarkers, including myocardial stress/injury-related (model 1; BNP, NT-proBNP, TnT, TnI), neurohormone-related (model 2; MR-proADM, AVP, Peptide), myocardial remodeling-related (model 3; sST2, Gal-3, MMP, GDF-15), inflammatory-related (model 4; CRP, IL-6, TNF-α, PCT), and renal function injury-related (model 5; Cys C, NGAL) biomarkers. Importantly, the AUC scores of the four genes’ combinations ranked first within the training cohort and test cohort (Figure S7), suggesting the superior prediction ability of our identified hub genes. The significant up-regulation patterns of four hub genes in HF were observed in the training cohort (*p* < 0.001, Figure 5B) and test cohort (*p* < 0.001, Figure 5E). Moreover, correlation analysis further revealed the close relationships between the expression levels of four hub genes. As shown in Figure 5C, *COL14A1* was significantly closely related to *OGN*, *SFRP4*, and *MFAP4* (R > 0.6, *p* < 0.001). Additionally, *SFRP4* highly correlated to *MFAP4* (R = 0.62, *p* < 0.001). The close expression correlation patterns of four hub genes were consistently verified in the test cohort (Figure 5F).

### 3.7. Distinct Cell-Specific Expression Patterns of Hub Genes in HF via scRNA-seq Analysis

**Figure 6:**
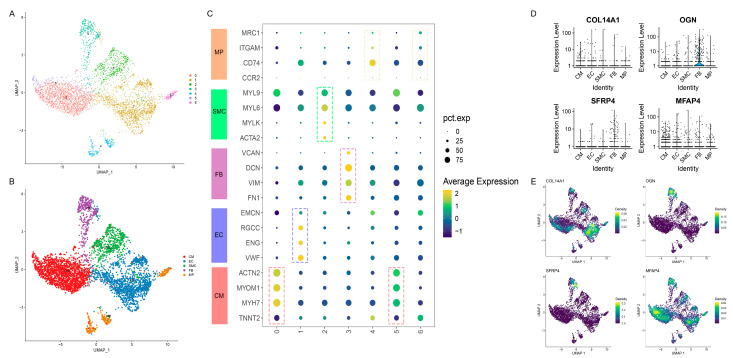
ScRNA-seq analysis uncovered the cell-specific expression patterns of hub genes in HF. (**A**) UMAP clustering of 4221 cells isolated from hearts under HF condition. (**B**) Annotation on clusters using specific gene markers of CM, EC, FB, SMC, and MP. (**C**) Seven clusters were annotated as five cell populations (CM, EC, FB, SMC, and MP). Dotted box indicates markers of five cell types. (**D**) Expression levels of hub genes in five cell populations. (**E**) Density estimation of hub genes in five cell populations. CM, cardiomyocyte; EC, endothelial cell; FB, fibroblast; SMC, smooth muscle cell; MP, macrophage.

To further explore the cell-specific expression of four hub genes in HF, we first conducted scRNA-seq analysis on GSE121893 to identify and characterize the cell populations in HF. The pre-processing procedure of scRNA-seq analysis, including quality control, data normalization, and dimensional reduction, is shown in Figure S8. Subsequently, the cells derived from six patients with HF were clustered into seven distinct clusters via the UMAP algorithm (Figure 6A). We then classified the seven clusters into five main cell types in HF (Figure 6B), including CMs, ECs, FBs, SMCs, and MPs, leveraging their specific molecular features (Figure 6C). Interestingly, we annotated clusters 4 and 6 as MPs, whereas distinct expression patterns of these clusters were observed. Cluster 4 is reflected with a higher *CD74*, *ITGAM*, and *MRC1* expression. However, their relatively lower expression was shown in cluster 6, suggesting that two cardiac MP populations are separately activated in the early stage and late stage. As depicted in Figure S9, we also evaluated the AUCell scores of the molecular features in the cell population, which notably differentiated five cell types. Next, we investigated the cell-specific expression patterns of four hub genes. The violin plots demonstrated that *OGN* and *SFRP4* were highly expressed in FBs (Figure 6D). However, the expression patterns of *COL14A1* and *MFAP4* showed no apparent differences among the five cell groups. Accordingly, the Nebulosa approach was implemented to recover the expression sparsity of hub genes from noisy signals in UMAP space. As shown in Figure 6E, we observed the clear expression patterns of four hub genes in five cell populations, in which *OGN* and *SFRP4* were mainly enriched in FBs, *COL14A1* in ECs, and *MFAP4* in CMs. Collectively, we uncovered the specific cellular compositions and expression patterns of hub genes in the context of HF.

### 3.8. GSVA and GSEA Revealed the Activated and Suppressed Pathways Co-Regulated by Hub Genes in HF

**Figure 7:**
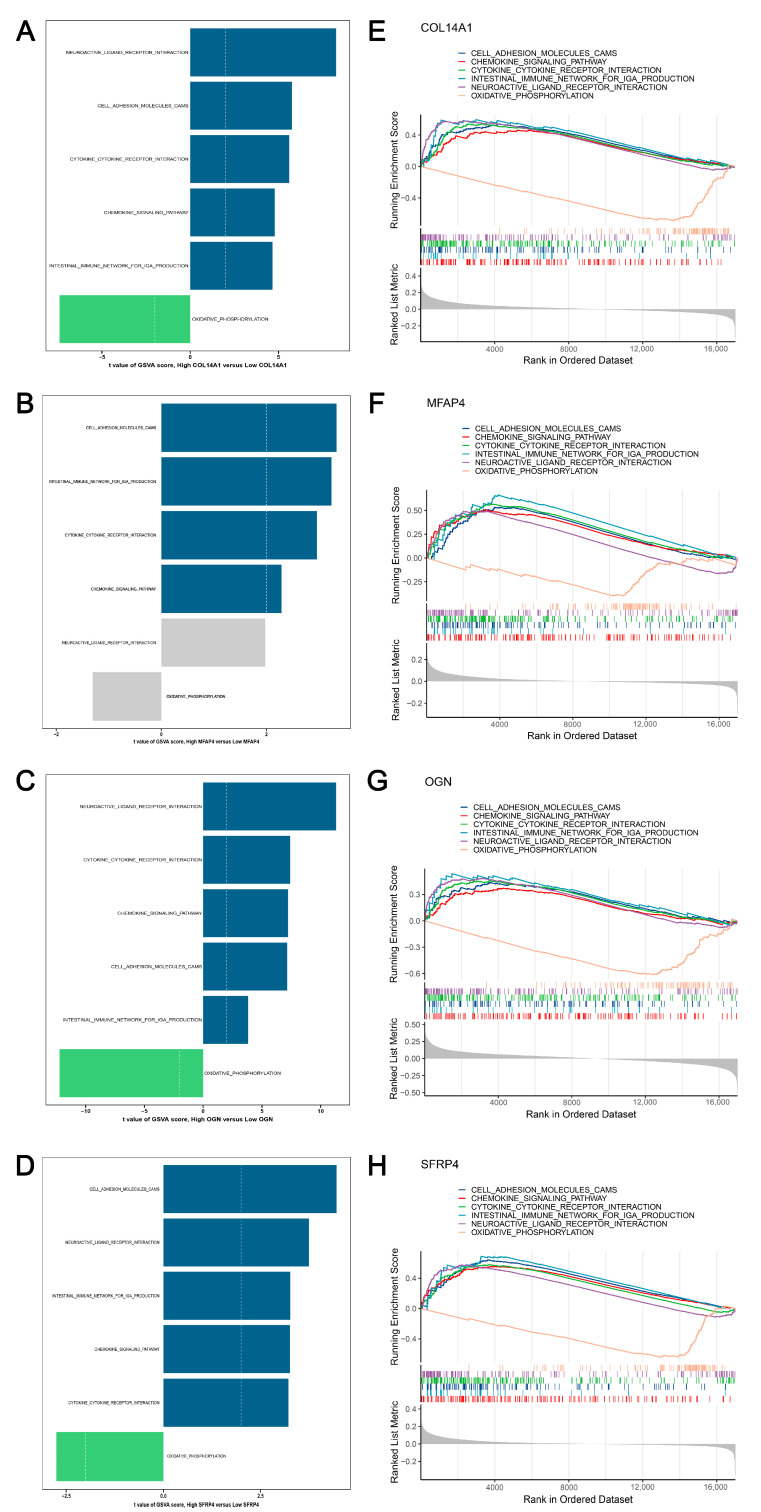
GSVA and GSEA of low and high expressions of hub genes involved in HF. (**A**) GSVA of *COL14A1*. (**B**) GSVA of *MFAP4*. (**C**) GSVA of *OGN*. (**D**) GSVA of *SFRP4*. (**E**) Selected top KEGG terms using GSEA of *COL14A1*. (**F**) Selected top KEGG terms using GSEA of *MFAP4*. (**G**) Selected top KEGG terms using GSEA of *OGN*. (**H**) Selected top KEGG terms using GSEA of *SFRP4*. Dotted line (**A**–**D**) suggests that the t value is greater than or less than 2.

To reveal the underlying pathways co-regulated by four hub genes, we performed GSVA and GSEA using 186 annotated KEGG pathways on the expression profiles of HF. First, we implemented GSVA on the training cohort and compared the GSVA scores of pathways between the low expression and high expression of each hub gene, respectively. The significantly activated pathways (t > 2, *p* < 0.05) and suppressed pathways (t < 2, *p* < 0.05) via GSVA were shown in Figure 7A–D and Figure S10A–D. Second, GSEA was performed on each gene within the training and test cohorts. As shown in Figure 7E–H, we observed that the chemokine signaling pathway, intestinal immune network for IgA production, cell adhesion molecules, cytokine–cytokine receptor interaction, and neuroactive ligand receptor interaction were significantly activated (NES > 1, *p* < 0.05, FDR < 0.25) in the training cohort. And oxidative phosphorylation was significantly inactivated (NES < 1, *p* < 0.05, FDR < 0.25) in the training cohort. These six significant GSEA-enriched pathways of hub genes in the training cohort were also validated in the test cohort (Figure S10). Third, we took the intersection between GSVA and GSEA of each hub gene (Figure S11). After comprehensively comparing the results from GSVA and GSEA, we suggested that cell adhesion molecules, the chemokine signaling pathway, cytokine–cytokine receptor interaction, and oxidative phosphorylation might be downstream mechanisms co-regulated by four hub genes in HF progression. Additionally, we implemented GSVA and GSEA analyses of these shared mechanisms in HF compared to control cases. Importantly, the evident activation of cell adhesion molecules, the chemokine signaling pathway, and cytokine–cytokine receptor interaction was observed in HF, but oxidative phosphorylation was inhibited (Figure S12). We subsequently conducted BN inference on the pathways mentioned above, and the predicted GRNs were visualized in Figure S13. The four hub genes showed higher expressions, more edges, and strengths in GRNs, suggesting their critical roles in regulating these pathways.

### 3.9. BNC2 and MEOX2 Were TFs Actively Targeting Hub Genes

**Figure 8:**
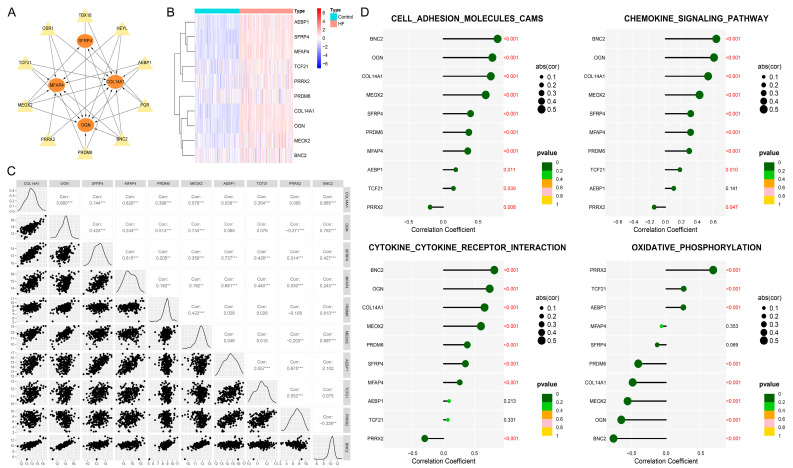
Interactions between TFs and hub genes-related pathways. (**A**) Hub gene–TF regulatory network: Circle symbolizes hub genes and triangle for TFs. (**B**) Heatmap showing the expression patterns of TFs and hub genes in HF and control groups. (**C**) Correlation heatmap of TFs and hub genes expressed in HF. (**D**) Lollipop plots showing the correlations between TF expression levels and ssGSEA scores of hub genes-related pathways. **, *p* < 0.01; ***, *p* < 0.001.

On the basis of the TRRUST v2 database, we enrolled the top 10 TFs (*OSR1*, *PRDM6*, *AEBP1*, *TBX18*, *MEOX2*, *BNC2*, *HEYL*, *TCF21*, *PRRX2*, and *PGR*) targeting hub genes (Table S10). A total of six TFs (*AEBP1*, *TCF21*, *PRRX2*, *PRDM6*, *BNC2*, and *MEOX2*) that increased in HF within the training and test cohorts (Figures S14 and S15) were regarded as key TFs that were activated in HF progression. The regulatory network involving four hub genes and six key TFs is shown in Figure 8A. Notably, *BNC2* and *MEOX2* showed regulatory interactions with four hub genes. The clustering heatmap then demonstrated the differential expression patterns of six key TFs and four hub genes in the HF and control groups (Figure 8B), in which the expressions of *AEBP1*, *TCF21*, and *PRRX2* were similar to those of *SFRP4* and *MFAP4*. *PRDM6*, *BNC2*, and *MEOX2* showed expression patterns similar to those of *COL14A1* and *OGN*. Figure 8C further showed the correlation patterns of 10 key TFs and four hub genes in HF, in which *BNC2* and *MEOX2* were significantly correlated to hub genes. We then quantified the enrichment levels of cell adhesion molecules, the chemokine signaling pathway, cytokine–cytokine receptor interaction, and oxidative phosphorylation in the HF group using ssGSEA. To evaluate whether the TFs could actively or negatively affect the hub genes-regulated pathways, we further related the expressions of key TFs to the ssGSEA enrichment scores of pathways. Figure 8D illustrates the significant positive relationships between two TFs (*BNC2*, *MEOX2*) and three pathways (including cell adhesion molecules, the chemokine signaling pathway, and cytokine–cytokine receptor interaction). However, oxidative phosphorylation was negatively correlated to *BNC2* and *MEOX2*, which indicated that these TFs inhibited oxidative phosphorylation. Notably, *PPRX2* exhibited a distinct correlation pattern compared to other key TFs, which was positively correlated with oxidative phosphorylation but negatively related to cell adhesion molecules, the chemokine signaling pathway, and cytokine–cytokine receptor interaction, suggesting that *PPRX2* might be a TF that suppressed hub genes in HF.

### 3.10. Elevated Infiltration Levels of Effector Memory CD4+ T Cells Were Highly Related to Hub Genes

**Figure 9:**
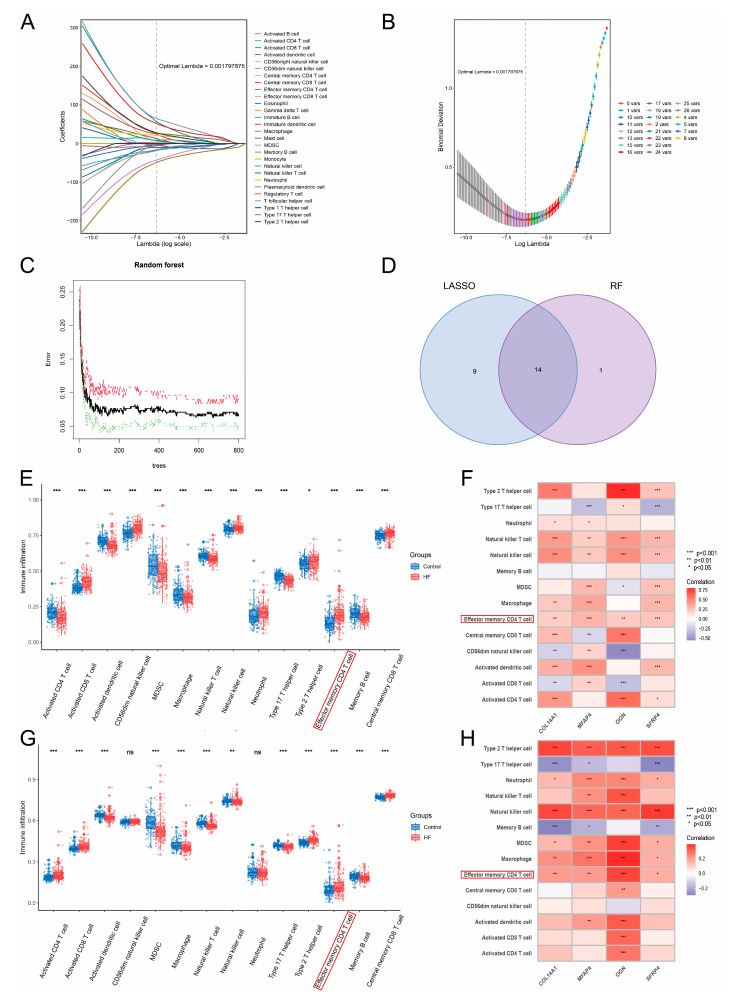
Exploring the immune landscape of HF and hub gene-related immune infiltration cells. (**A**) Lasso coefficient profiles of 28 immune infiltration cells. (**B**) Selection of optimal penalization coefficient (λ) under 10-fold cross-validation. (**C**) The effect of tree number on error variation of RF. Green and red stand for the error rate of the control and HF groups, respectively, and black represents the overall error rate. (**D**) Integration of Lasso and RF identified 14 key immune infiltration cells of HF. (**E**) Comparisons of 14 key immune infiltration cells between the HF and control groups in the training cohort. (**F**) Correlation analysis among 14 key immune infiltration cells and hub genes in the training cohort. (**G**) Comparisons of 14 key immune infiltration cells between the HF and control groups in the test cohort. (**H**) Correlation analysis among 14 key immune infiltration cells and hub genes in the test cohort. Effector memory CD4+ T cells are highlighted in the red frame, indicating that the infiltration of effector memory CD4+ T cells elevates in HF and is positively correlated to hub genes. ns, not statistically significant; *, *p* < 0.05; **, *p* < 0.01; ***, *p* < 0.001.

In the previous analysis, we observed that the expressions of hub genes may co-regulate two significantly activated immune-related pathways in HF, including the chemokine signaling pathway and cytokine–cytokine receptor interaction. Accordingly, we wondered whether the immune landscape altered and could further exert certain functions in HF. First, we performed ssGSEA to quantify the infiltration levels of 28 immune cell types between the HF and control groups within the training and test cohorts. We then carried out Lasso and RF to perform feature selection on the infiltration profiles of 28 immune cell types to identify the key types, respectively. Under the optimal λ (0.001797875), Lasso obtained 23 key immune cell types (Table S11) with regression coefficients larger than zero (Figure 9A,B). RF constructed with 586 decision trees retained 15 immune cell types (Table S12) with a mean decrease in Gini larger than zero (Figure 9C). A set of 14 immune cell types (activated CD4+ T cell, activated CD8+ T cell, activated dendritic cell, CD56dim natural killer cell, MDSC, macrophage, natural killer T cell, natural killer cell, neutrophil, Type 17 T helper cell, Type 2 T helper cell, effector memory CD4+ T cell, memory B cell, central memory CD8+ T cell) overlapping between Lasso and RF was considered to contain the key immune cell types of HF. Within the training and test cohorts, we demonstrated the elevated infiltrations of activated CD8+ T cells, Type 2 T helper cells, and effector memory CD4+ T cells in HF (Figure 9D,F). We also noticed that the infiltrations of natural killer cells and effector memory CD4+ T cells positively related to expressions of hub genes in the training and test cohorts (Figure 9E,G). Effector memory CD4+ T cells showed significantly up-regulated infiltrations and positive correlations with hub genes in HF, suggesting its crucial role involved in immune-related pathways regulated by hub genes.

### 3.11. Small-Molecule Agents Targeting Active TFs and Hub Genes Could Serve as Candidate Drugs for Alleviating HF

A set of 78 small-molecule agents potentially targeting two key TFs or four hub genes was identified via the Enrichr database by accessing DSigDB (Table S13). We then identified captopril (targeting *BNC2*), aldosterone (targeting *MEOX2*), cyclopenthiazide (targeting *MEOX2*), estradiol (targeting *COL14A1*), tolazoline (targeting *COL14A1*), and genistein (targeting *SFRP4*) previously have been reported to treat or alleviate HF [64,65,66,67,68,69], as candidate therapeutic agents for HF. Notably, captopril and cyclopenthiazide belong to Angiotensin-Converting Enzyme Inhibitors (ACEIs) and thiazide diuretics, which have been put into clinical treatment of HF. Moreover, the therapeutic value of aldosterone antagonists in curving HF has been underscored recently. Given the protective effects of estrogens and phytoestrogens for alleviating HF, studies from animal models have demonstrated that genistein and estradiol could attenuate pathological hypertension in the progression of HF, suggesting their HF-related preventative roles. AutoDock Vina software (version 1.1.2) was subsequently performed to dock the six selected small-molecule agents with their targeted TFs (*BNC2*, *MEOX2*) or hub genes (*COL14A1*, *SFRP4*). Furthermore, we visualized the docking models with the lowest binding affinity and Root Mean Squared Error (RMSE) lower than 2 Å through PyMOL (Figure S16). The formation of hydrogen bonds that small-molecule agents bind to the amino acids of TFs or hub genes was also analyzed (Table 2, Figure S16), thus revealing the specific affinity patterns.

### 3.12. Plasma OGN Elevated in HF with Robust Diagnostic Value and Positive Causal Correlation to HF Risk

**Figure 10:**
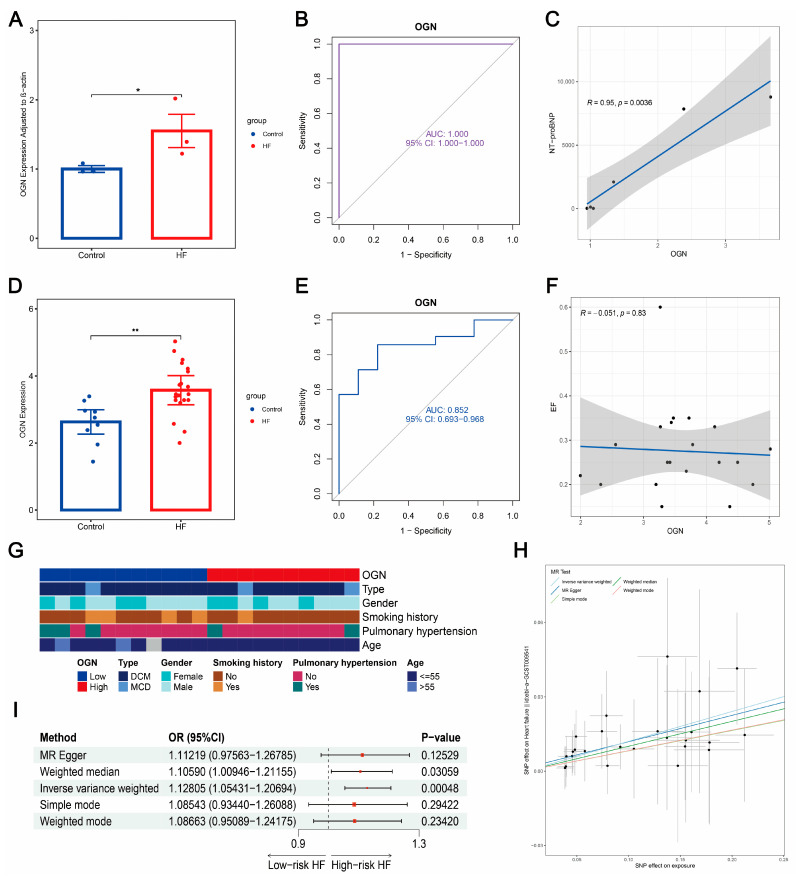
Evaluation of plasma *OGN* as a promising diagnostic biomarker of HF. (**A**) The qPCR-detected mRNA expression level of *OGN* (normalized by β-actin) in plasma of HF patients compared to control cases. (**B**) ROC curve of *OGN*. (**C**) Correlation between expressions of *OGN* and NT-proBNP. (**D**) The expressions of *OGN* in HF patients compared to control cases in GSE135055. (**E**) ROC curve of *OGN* in GSE135055. (**F**) Correlation between expressions of *OGN* and EF in GSE135055. (**G**) Heatmap displaying the expression patterns of *OGN* in groups of different clinical characteristics in GSE135055. (**H**) A scatterplot shows the positive association between the SNP effect on plasma *OGN* (x-axis) and HF (y-axis). (**I**) Forest plot indicating the estimated OR effects and *p* values generated from using five approaches on assessing the causal effect of plasma *OGN* on the risk of AMI. The size of red box indicates standard error. *, *p* < 0.05; **, *p* < 0.01.

To study whether the four hub genes could serve as plasma biomarkers for diagnosing HF, we conducted qRT-PCR analysis on plasma collected from three HF patients and three non-HF controls. As shown in Figure 10A, *OGN* was highly expressed in HF, elevating about 1.5 times compared to the control. Additionally, *OGN* demonstrated an extremely robust diagnostic value (AUC = 1) in distinguishing HF from control (Figure 10B). A positive correlation between *OGN* and NT-proBNP (R = 0.95, *p* = 0.0036) is shown in Figure 10C. Leveraging GSE135055 as an external validation cohort, we noted that in comparison with the control, the expressions of plasma *OGN* in patients with HF also significantly increased (Figure 10D). Moreover, the robust diagnostic power of *OGN* was again demonstrated (AUC = 0.852, Figure 10E). Meanwhile, no significant relationship between *OGN* and EF was observed (*p* = 0.83, Figure 10F). Figure 10G further showed the expression patterns of *OGN* among diverse groups of clinical characteristics, including HF type, gender, smoking history, hypertension, and age. Interestingly, we found that the expression of *OGN* may not fluctuate through these clinical factors (all *p* > 0.05), which were subsequently validated in the training and test cohorts (Figure S17). To investigate the potential of plasma *OGN* as a diagnostic biomarker benefiting from genetic variation, we performed a two-sample MR analysis based on large-scale GWAS data. We initially integrated the exposure data (plasma *OGN*) and outcome data (HF risk) to filter the overlapping significant SNPs. Following the three basic assumptions of the two-sample MR approach, we selected 25 significant SNPs as strong IVs to implement the analysis. As depicted in Figure 10H, we related the effect sizes of SNPs on plasma *OGN* and SNPs on HF, and there was an overall positive fitted relationship, suggesting the direct casual association between plasma *OGN* and HF. The forest plot and funnel plot of each SNP on HF can be found in Figure S18A,B, respectively. Using the IVW approach, which showed the highest statistical effectiveness among the five MR methods, we demonstrated that plasma *OGN* was positively correlated to the risk of HF with an estimated OR of 1.12805 (95% CI: 1.05431–1.20694, *p* < 0.05, Figure 10I). The positive causal relationship between plasma *OGN* and HF was also estimated via the other four MR approaches (Figure 10H), showing a consistent directional change (all OR > 1). Thus, we herein speculated that a higher concentration of plasma *OGN* corresponds to an increased risk of HF. To guarantee the reliability of this result, we conducted a series of sensitivity tests (heterogeneity, horizontal pleiotropy, and leave-one-out analyses). No heterogeneity and horizontal pleiotropy were observed (all *p* > 0.05, Table S14). In the leave-one-out analysis, the step-by-step removal of each SNP was not recognized to greatly influence the result (all SNP > 0, Figure S18C), indicating all incorporated SNPs achieved significant causality rather than the occurrence of a dominate SNP. In a word, these data suggested the great potential of *OGN* as a plasma biomarker for HF diagnosis and the evaluation of HF progression.

## 4. Discussion

HF remains high in incidence and mortality worldwide, and a novel approach is urgently needed to improve this situation. In the past few decades, HF has been proven to be a heterogeneous and progressive clinical syndrome that is reflected in its complex pathophysiology [6].

**Figure 11:**
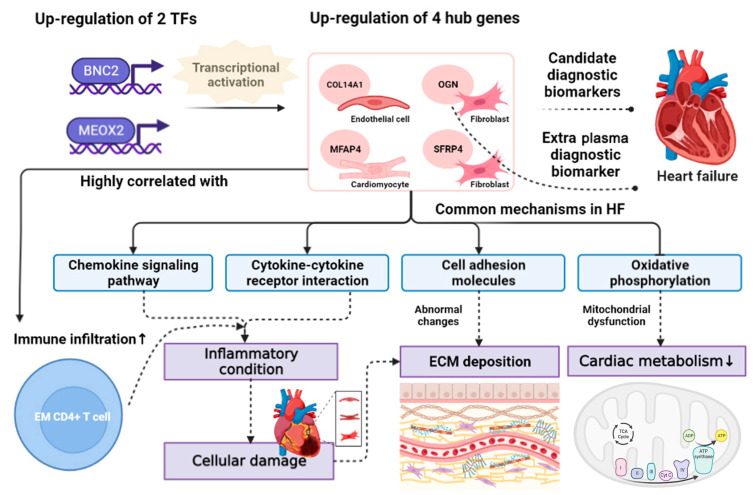
Schematic diagram illustrating the speculative pathogenic mechanisms of four hub genes that participated in HF.

Herein, we performed a comprehensive bioinformatics analysis on bulk RNA-seq and sc-RNA seq datasets from HF patients and non-HF controls. We briefly outlined our main findings shown in a schematic diagram (Figure 11). First, we identified 926 distinct DEGs (HF versus control), including 648 up-regulated genes and 278 down-regulated genes in HF. The KEGG pathway enrichment analysis then showed that DEGs were mainly involved in ECM and immune-related pathways (cytokine–cytokine receptor interaction, cell adhesion molecules, the T cell receptor signaling pathway, ECM–receptor interaction, complement and coagulation cascades). Impaired signaling through corresponding cytokine receptors accelerates myocardial apoptosis and tissue damage after acute cardiac stress, suggesting an adverse role of cytokine–cytokine receptor interactions in HF [70]. Furthermore, cell adhesion molecules play a crucial role in modulating cardiac inflammation and pathological cardiac remodeling by facilitating the recruitment of T-cells to the left ventricle, ultimately contributing to cardiac dysfunction of HF [71]. Excessive extracellular matrix remodeling has extensively been known to precipitate HF. Coagulation proteases orchestrate clotting-independent activation of protease-activated receptors (PARs) to modulate TGF-β1 signaling, influencing cardiac fibrosis in HF [72]. Notably, a large body of evidence has demonstrated the abnormal ECM and immune-related functions or mechanisms altered in HF development and progression.

Gene co-expression analysis is commonly used to identify gene modules linked to disease traits [73]. Next, we performed WGCNA and MEGENA gene co-expression analyses on DEGs. Leveraging the gene–pairwise correlation and agglomerative clustering, WGCNA allows the assignment of each gene to certain modules, which helps recognize the modules highly correlated to disease traits. We initially identified two crucial modules from WGCNA, including ME turquoise and ME blue modules. Of note, these two modules all displayed significant strong correlations to HF (R > 0.7, *p* < 0.05), and genes recognized in these modules showed positive associations between GS and MM. These results suggest that these two modules exhibited a strong linkage with HF, which could be used as functional gene sets of HF. Contrary to the traditional clustering by WGCNA, MEGENA is implemented based on divisive hierarchical clustering [74]. Therefore, more compact and functionally coherent modules could be screened out through MEGENA, and we recognized two large gene modules from MEGENA, including c1_2 and c1_6 modules. WGCNA and MEGENA could complement each other [74] and comprehensively capture gene modules strongly related to HF rather than rely on a single method. Four gene modules were obtained, including two WGCNA-identified gene modules (ME turquoise and ME blue modules) and two MEGENA-identified gene modules (c1_2 and c1_6 modules). More interestingly, we obtained two large intersections between the four identified gene modules, which were 248 genes shared by the ME turquoise and c1_6 modules and 234 genes common to the ME blue and c1_2 modules. Although WGCNA and MEGENA successfully seized these two preserved co-expression gene patterns associated with HF, their biological significance of HF remained uncertain. Therefore, we implemented enrichment analysis to annotate human disease, gene functions, and pathways to determine whether the HF-related terms are over-enriched or under-enriched in the two overlapping gene sets. Here, we selected gene sets with more HF-representative terms to guarantee the algorithmic and biological capture of HF characteristics. The DO, GO, and KEGG enrichment analyses demonstrated that 248 genes were mainly involved in diverse cardiovascular disorders and HF-related biological functions or pathways. In comparison, fewer HF-related enriched terms were shown in 234 genes. Consequently, we retained 248 genes and performed feature selection via a machine learning-based integration pipeline under the LOOCV framework. A total of 134 feature genes were selected using the Enet (alpha = 0.1) model with the highest AUC values in the training and test cohorts. Together with the PPI network establishment and five ranking algorithms of Degree, MNC, MCC, EPC, and DMNC, we further obtained 10 pivotal genes, including *KIT*, *SLC6A1*, *SLC6A4*, *COL14A1*, *MME*, *OGN*, *SFRP4*, *CD1C*, *MFAP4*, *HTR2B*. Most of these pivotal genes’ activations have been reported to contribute to HF, as summarized in Table S15. ROC curve analysis then identified four key genes (*COL14A1*, *OGN*, *SFRP4*, *MFAP4*) performed with robust AUC scores (AUC > 0.85) in both the training and test cohorts. The up-regulation patterns of four key genes in HF and their positive correlation patterns were observed within the training and test cohorts. Leveraging sc-RNA seq analysis, we uncovered the cellular composition of failed hearts, mainly including cardiomyocytes, endothelial cells, fibroblasts, smooth muscle cells, and macrophages. Moreover, distinct cell-specific expression patterns were revealed in which *OGN* and *SFRP4* were mainly expressed in cardiac fibroblasts, *COL14A1* was mainly expressed in cardiac endothelial cells, and *MFAP4* was expressed in cardiomyocytes.

Of these four key genes, Collagen Type XIV Alpha 1 Chain (*COL14A1*) has previously been reported to affect arterial remodeling [75] and showed up-regulation mRNA and protein levels in the ischemic heart of HF patients [76]. *Col14a1* deficiency would contribute to defective ventricular morphogenesis and chaotic collagen fibril organization in mice, thereby indicating its maintenance of structural integrity during early heart development [77]. Given the specific expression pattern of *COL14A1* in endothelial cells uncovered by sc-RNA analysis, we speculate that the close association of *COL14A1*-activated endothelial cells and dysfunction in collagen constituents contributes to myocardial hypertrophy of HF progression. Osteoglycin (*OGN*), which encoded a class III small leucine-rich proteoglycan (SLRP) member, was first discovered to act as a key regulator in rat left ventricular mass (LVM) through modulating the TGF-β pathway [78]. It was also proposed that *OGN* might be involved in the regulation of cardiac dysfunction and adverse remodeling after myocardial infarction in HF [79]. Moreover, *OGN* was reported to modulate fibrosis and inflammation, resulting in diastolic dysfunction shown in hypertensive heart disease [80]. More recently, Fang et al. have revealed the specific mechanism of *Ogn* in myocarditis mice by which its silencing suppressed the Wnt signaling pathway, thus inhibiting myocardial fibrosis proliferation [81]. The close linkage between activated cardiac fibroblast and adverse outcomes following HF (fibrosis, remodeling, and dysfunction) has been extensively expounded [82]. Therefore, it is reasonable to deduce the expression of *OGN* active in cardiac fibroblasts in the context of HF, leading to myocardial fibrosis and remodeling post-HF. Microfibril-associated glycoprotein 4 (*MFAP4*) has been proven to regulate calcium-dependent cell adhesion or intercellular interactions, leading to inflammation and fibrosis, which was previously verified to engage in remodeling-related diseases such as liver fibrosis, atherosclerosis, and arterial injury stimulated remodeling [83,84]. Apart from the close association between *MFAP4* and remodeling-related disorders, it was also shown that *MFAP4* could also serve as a candidate biomarker for cardiovascular diseases such as HF [84]. For instance, clinical cohort-based research demonstrated that the protein levels of *MFAP4* were significantly increased in serum derived from HF patients compared to control cases [85]. Mechanistically, *Mfap4* deletion could attenuate cardiac fibrosis and ventricular arrhythmias, suggesting its potential as a therapeutic target in HF prevention [86]. Additionally, Dorn et al. showed that *Mfap4* knockout affected pressure overload-induced cardiac remodeling, leading to elevated cardiac hypertrophy and exacerbating cardiac function [87]. According to our previous analysis, *MFAP4* was identified to be specifically expressed in cardiomyocytes, which largely implies its significant activation in directly exacerbating hypertrophy of cardiomyocytes in the condition of HF. Secreted frizzled-related protein 4 (*SFRP4*) has been reported to be expressed in human ventricular myocardium and correlates with apoptosis-related gene expression [88]. Zeng et al. showed that the increased *Sfrp4* in mice myocardial infarction model and knockout of *Sfrp4* could protect against myocardial ischemia and reperfusion injury through attenuating apoptosis of cardiomyocytes [89]. Additionally, evidence from the rat heart ischemic model showed that *Sfrp4* recombinant protein injection could exert a cardio-protective effect and improve the cardiac function of the ischemic heart, which suggested the therapeutic potential of *SFRP4* in humans [90]. Regarding the evident expression of *SFRP4* in cardiac fibroblasts of failed hearts, we mechanically speculated that elevated *SFRP4* affects myocardial fibrosis and remodeling post-HF. Ji et al. found that plasma *SFRP4* concentrations were increased in patients with coronary artery disease (CAD) and acted as an independent factor [91]. Altogether, findings from prior experimental and clinical evidence have indicated the great potential of four hub genes in developing ECM fibrotic, immune, or cardiac remodeling-related biomarkers and therapeutic molecular targets of HF.

Subsequently, we integrated GSVA and GSEA algorithms to explore the shared pathways regulated by the four hub genes in HF. Three significantly activated pathways (including cell adhesion molecules, the chemokine signaling pathway, and cytokine–cytokine receptor interaction) were co-identified through the combination of GSVA and GSEA. In contrast, oxidative phosphorylation was identified as a common suppressed pathway. Notably, the induction of ECM remodeling, inflammatory cytokines, and chemokines have been proven to contribute to the pathogenesis of HF, such as adverse remodeling, myocardial injury, myocardial fibrosis, and dysfunction [70,71,92,93]. Moreover, it was shown that impaired mitochondrial respiratory and decreased oxidative phosphorylation were detected in the hearts of HF patients [94]. More strikingly, we observed the same phenomenon through GSVA and GSEA algorithms: cell adhesion molecules, chemokine signaling pathway, and cytokine–cytokine receptor interaction were active, whereas oxidative phosphorylation was inhibited in HF compared to control. We noticed that four hub genes participated in diverse pathogenic pathways involving ECM, immune factors, and oxidative phosphorylation, suggesting the pathophysiologic heterogeneity of HF. Given the close interplays between these pathogenic pathways in HF progression, we speculated that the continuous induction of chemokines or cytokines stimulated the formation of inflammatory conditions in HF that promoted ECM deposition and impaired oxidative phosphorylation capability, thereby exerting an adverse effect on cardiac function (Figure 11). Regarding the upstream regulatory mechanism that could activate the transcription of hub genes, the transcription factors were predicted using the TRRUST v2 database. After systemically exploring the correlation patterns between the up-regulated TFs in HF and the hub gene-regulated pathogenic pathways, we identified *BNC2* and *MEOX2* as upstream regulators to activate the transcription of hub genes and drive the pathogenic pathways. Basonuclin 2 (*BNC2*), a zinc finger TF, was recently identified as a core TF essential for myofibroblastic activation in fibrosis, causing ECM deposition during fibrogenesis [95]. Mesenchyme Homeobox 2 (*MEOX2*) has previously been reported to regulate the proliferation, differentiation, and migration of vascular endothelial cells and cardiomyocytes [96,97]. *BNC2* and *MEOX2* are closely linked with cardiac functions and may contribute to HF-related pathogenic pathways, which could be confident upstream regulators during HF progression. Taken together, we uncovered a comprehensive mechanism of hub genes involved in HF, which was a compact regulatory interaction of TFs–hub genes–pathways: The up-regulations of TFs (*BNC2* and *MEOX2*) activate the transcriptions of four hub genes (*COL14A1*, *OGN*, *MFAP4*, *SFRP4*) and subsequently drive the activation or suppression of downstream signaling pathways (Figure 11). The interplays between activated pathways related to immune factors and ECM and the suppressed oxidative phosphorylation pathway may further cause abnormal cardiac functions, thereby promoting the development and progression of HF.

Given that the immunity-related pathways involving chemokine and cytokine were observed in hub genes, we wondered which main type of immune cell altered in HF could regulate or participate in these pathways. Leveraging the ssGSEA algorithm, we quantified the infiltration levels of 28 types of immune cells based on the gene expression profiles. Combining with Lasso and RF models, we then obtained a total of 14 key immune cell types with HF (activated CD4+ T cell, activated CD8+ T cell, activated dendritic cell, CD56dim natural killer cell, MDSC, macrophage, natural killer T cell, natural killer cell, neutrophil, Type 17 T helper cell, Type 2 T helper cell, effector memory CD4+ T cell, memory B cell, and central memory CD8+ T cell). Together with expression and correlation analyses on the training and test cohorts, we finally suggested effector memory CD4+ T cell, which demonstrated both increased infiltrations in HF and positive relationships with hub genes, as an essential immune cell type that participated in the chemokine signaling pathway and cytokine–cytokine receptor interaction with hub genes. Preliminary evidence has shown that the infiltrations of effector memory CD4+ T cells caused cardiac fibroblast activation and subsequent fibrosis, influencing pressure-overload-induced HF [98]. This result motivated us to consider that the elevated infiltrations of effector memory CD4+ T cells could activate the chemokine signaling pathway and cytokine–cytokine receptor interaction—potentially by the recruitment of chemokines and cytokines.

Considering that four hub genes (*COL14A1*, *OGN*, *MFAP4*, and *SFRP4*) and two key TFs (*BNC2* and *MEOX2*) may serve as potential therapeutic targets of HF, several small-molecule agents targeting hub genes were predicted. Then, molecular docking showed that the close binding patterns between ligands (small-molecule agents) and receptors (hub genes or TFs), including captopril-targeting *BNC2*, aldosterone-targeting *MEOX2*, cyclopenthiazide-targeting *MEOX2*, estradiol-targeting *COL14A1*, tolazoline-targeting *COL14A1*, and genistein-targeting *SFRP4*. We noticed that the estimated RMSE from the docked model was lower than 2 Å, indicating the high credibility of molecular docking. Furthermore, we collected the high-quality structures of receptors and ligands, which largely guarantee the accuracy of molecular docking. Using the molecular docking technique, we illustrated the treatment potential of these TFs and genes. Although the effectiveness of these molecules as drug targets in curing HF was demonstrated from an in-silico perspective, further animal studies and clinical trials are needed. Notably, these small-molecule agents were previously reported to cure or alleviate HF symptoms [64,65,66,67,68,69]. Therefore, the identified small-molecule agents may reverse the over-expression levels of two hub genes (*COL14A1*, *SFRP4*) and two TFs (*BNC2*, *MEOX2*) in HF, thus inhibiting the regulatory network of TFs–hub genes–pathways and then alleviating HF progression.

In the previous analysis, we illustrated the great potential of four hub genes as molecular diagnostic biomarkers and therapeutic targets for HF. To further investigate whether the four hub genes could serve as extra plasma biomarkers of HF for clinic applications, we collected six whole blood samples derived from three HF patients and three control cases. Then, the qRT-PCR results showed that *OGN* was highly expressed in the plasma of HF patients compared to the control cases. Additionally, *OGN* demonstrated robust diagnostic accuracy in HF recognition and a significantly positive correlation to NT-proBNP concentration. The elevated pattern and robust diagnostic power of plasma *OGN* were subsequently verified in an external cohort, including 21 HF and nine control cases. Some clinical factors, particularly gender and age, have been reported to cause differences in the detection of HF biomarkers [99]. For instance, gender-related differences in Cardiac Troponin (cTn) values have been evident in patients with HF [100] with higher values commonly shown in males. However, most clinicians barely consider these factors when using these biomarkers [100]. Thus, more effort should be put into investigating the interference on diagnostic values of biomarkers, considering the underlying differences brought by the confounding factors. Herein, we deduced that the successful clinically used biomarker should be stably detectable for reflecting disease rather than being disturbed via different characteristics of patients. Accordingly, we wondered whether the expression levels of *OGN* varied in different clinical characteristics, mainly including HF type, gender, and age. Notably, no significant expressions of *OGN* differed in groups of these clinical factors. This result suggests that *OGN* may serve as a stable biomarker for HF detection with limited affection brought by the confounding factors such as gender and age. Given the promising application of plasma OGN in discerning HF, we also studied the causal association of plasma *OGN* and the risk of HF from the genetic insight using GWAS datasets. We used the SNPs as IVs for a two-sample MR study based on the exposure (plasma *OGN*) and outcome (HF) GWAS data. As a result, we found that plasma *OGN* was significantly causally related to the increased risk of HF with an overall OR estimated larger than one measured by five MR approaches. Strikingly, the genetic variant in *OGN* was proven to promote HF occurrence on the large-scale GWAS data, which largely sustained the great potential of *OGN* as an HF biomarker. Thus, these results demonstrated that *OGN* could serve as a plasma detective biomarker that is more indicative of HF patients and HF progression.

Nevertheless, some limitations remain in our present study. First, these findings were mainly generated from an in silico analysis of transcriptional datasets and a need for in-vitro confirmative studies. We are pushing ahead with a larger-scale investigation by integrating more in silico and in vitro datasets related to HF. However, it remained an effective means to rapidly explore the candidate diagnostic biomarkers and underlying pathogenic mechanisms of HF, thus providing meaningful and constructive references for understanding HF. Second, we detected the up-regulated *OGN* expressions in the plasma of three HF patients compared to three control cases. The sample sizes were relatively limited. Further validation should be conducted within a prospective and larger multi-center collaboration, which guarantees the credibility of *OGN* in diagnostic applications. Given our understanding of the biomarkers-regulated pathogenic mechanisms in HF, more in-depth molecular studies, such as genetic knockout and pharmacological exploration on cell or animal models, are needed to substantiate our work.

## 5. Conclusions

Four hub genes (*COL14A1*, *OGN*, *MFAP4*, and *SFRP4*) were identified as candidate molecular biomarkers for the diagnosis of HF. Subsequently, the pathogenic mechanisms of hub genes were expounded. The up-regulation of two key TFs (*BNC2* and *MEOX2*) prompted the expression of hub genes and further activated several ECM and immune-related signaling pathways, including cell adhesion molecules, the chemokine signaling pathway, and cytokine–cytokine receptor interaction while inhibiting the oxidative phosphorylation pathway. Moreover, elevated infiltrations of effector memory CD4+ T cells in HF were closely related to the expressions of hub genes. They may contribute to activating the chemokine signaling pathway and cytokine–cytokine receptor interaction, leading to the inflammatory condition of HF. Six small-molecule agents, including captopril, aldosterone antagonist, cyclopenthiazide, estradiol, tolazoline, and genistein, were predicted in silico to exhibit interactions with two key TFs (*BNC2* and *MEOX2*) and two hub genes (*COL14A1* and *SFRP4)*. These agents could be developed into therapeutic drugs to treat HF by curbing the up-regulation of these TFs or hub genes. Finally, our in vitro study on clinical specimens suggested that *OGN* could serve as a plasma diagnostic biomarker of HF with robust diagnostic accuracy and positive correlation with NT–proBNP concentration. Two-sample MR analysis further demonstrated the positive causal association between plasma *OGN* and the increased risk of HF. Altogether, our study proposes *OGN* as a candidate diagnostic biomarker of HF and provides novel insight into the pathogenesis of HF.

## Figures and Tables

**Table 1:** Information of HF-related datasets enrolled in study.

Accession	Sample Source	Sequencing Type	Control Samples	HF Samples	Dataset Usage
GSE141910	left ventricle	RNA-seq	166	200	Training dataset
GSE57338	left ventricle	Array	136	177	Testing dataset
GSE42955	left ventricle near the apex	Array	5	24	Testing dataset
GSE135055	left ventricle	RNA-seq	9	21	External validation dataset

**Table 2:** Molecular docking results of drugs with targets.

Drugs	Targets	Affinity (kcal/mol)	Bond	Protein’s Residues
Captopril	*BNC2*	−3.9	H-bond	GLN-199
Aldosterone	*MEOX2*	−7.1	H-bond	GLU-203 LYS-241
Cyclopenthiazide	*MEOX2*	−5.7	H-bond	THR-192 ASN-237
Estradiol	*COL14A1*	−8.2	H-bond	ARG-171 VAL-930
Tolazoline	*COL14A1*	−5.9	H-bond	ASP-1148
Genistein	*SFRP4*	−3.8	H-bond	GLU-127 ARG-262

## Data Availability

Data used in this study, including GSE141910, GSE57338, GSE42955, GSE121893, and GSE135055 can be downloaded from the GEO database (https://www.ncbi.nlm.nih.gov/geo/, accessed on 30 November 2022).

## Supplementary Materials

The following supporting information can be downloaded at https://www.mdpi.com/article/10.3390/biom14020179/s1, Table S1: The clinical information of HF patients and control cases in GSE141910; Table S2: The clinical information of HF patients and control cases in GSE57338; Table S3: The clinical information of HF patients and control cases in GSE42955; Table S4: The clinical information of HF patients and control cases in GSE135055; Table S5: The clinical information of HF patients and control cases of our in-house cohort; Table S6: The primer sequences of hub genes and β-actin; Table S7: Detailed information of 926 DEGs; Table S8: Genes in WGCNA-identified modules; Table S9: Genes in MEGENA-identified modules; Table S10: Top 10 predicted TFs with hub genes; Table S11: Lasso-selected immune cell types; Table S12: RF-selected immune cell types; Table S13: Predicted drugs targeting TFs and hub genes; Table S14: Sensitivity analysis of two-sample MR; Table S15: Summary of biological functions of ten pivotal genes; Figure S1: Workflow of the study; Figure S2: PCA plot of merging test cohort. Figure S3: Construction of weighted gene co-expression network on DEGs. Figure S4: Systematic screening of pivotal genes using five algorithms of cyto-Hubba. Figure S5: ROC curve analysis to evaluate the diagnostic accuracies of pivotal genes. Figure S6: The diagnostic accuracies of four hub genes in the test cohort. Figure S7: Comparison of AUC scores on our identified biomarkers with current biomarkers; Figure S8: Quality control and dimension reduction in sc-RNA seq data. Figure S9: AUCell scores of different gene markers in each cell population. Figure S10: GSVA and GSEA of hub genes. Figure S11: The activated and suppressed pathways are shared by hub genes; Figure S12: GSVA and GSEA of shared pathways by hub genes within the control and HF cases. Figure S13: The Bayesian networks show the GRN of common pathways regulated by hub genes. Figure S14: Expression levels of 10 predicted TFs in HF compared to the control group in the training cohort. Figure S15: Expression levels of 10 predicted TFs in HF compared to the control group in the test cohort. Figure S16: Molecular docking of candidate drugs with TFs and hub genes. Figure S17: Expression patterns of OGN across diverse clinical characteristics of HF patients. Figure S18: Two-sample MR analysis.
