# Supplementary figures and images for: Identifying OGN as a Biomarker Covering Multiple Pathogenic Pathways for Diagnosing Heart Failure: From Machine Learning to Mechanism Interpretation
Source: Biomolecules. 2024 Feb 2;14(2):179. doi: 10.3390/biom14020179 (PMC10886937; doi:10.3390/biom14020179)

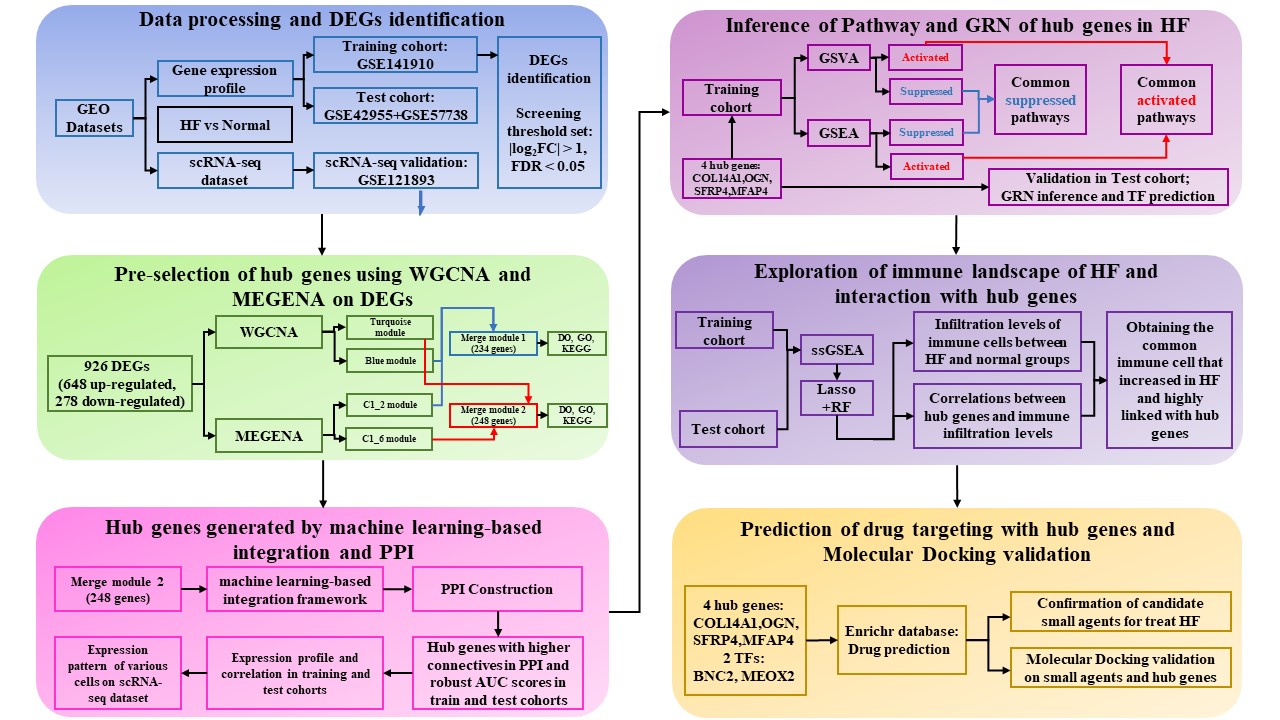

Supplement: Supplementary file 1 [file biomolecules-14-00179-s001.zip › Supplementary Figure S1.jpg]

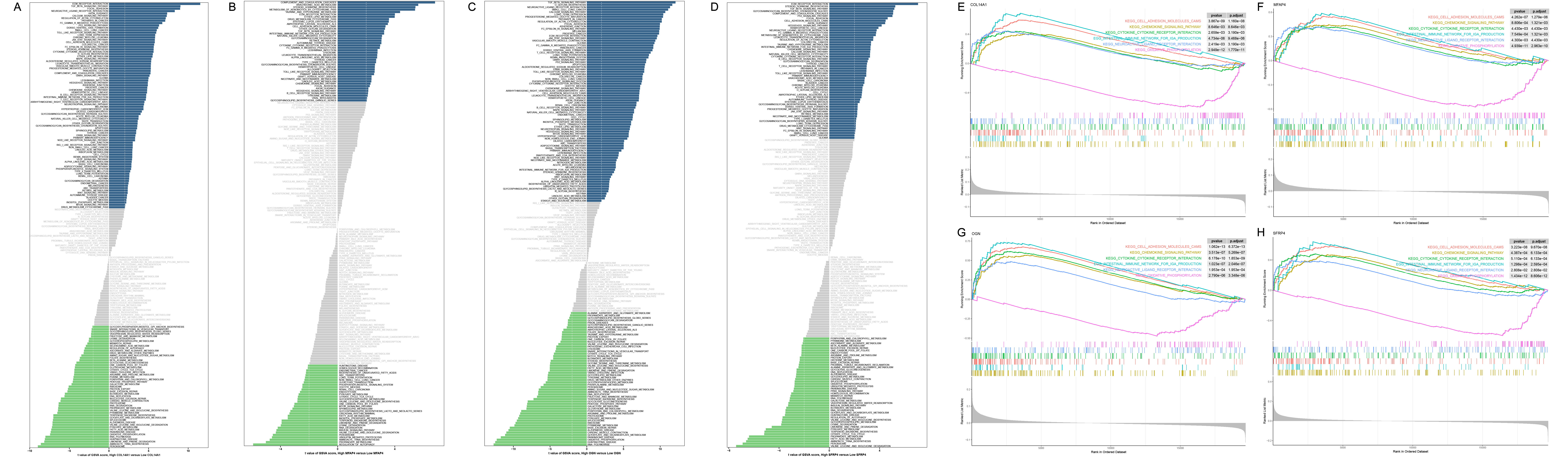

Supplement: Supplementary file 1 [file biomolecules-14-00179-s001.zip › Supplementary Figure S10.jpg]

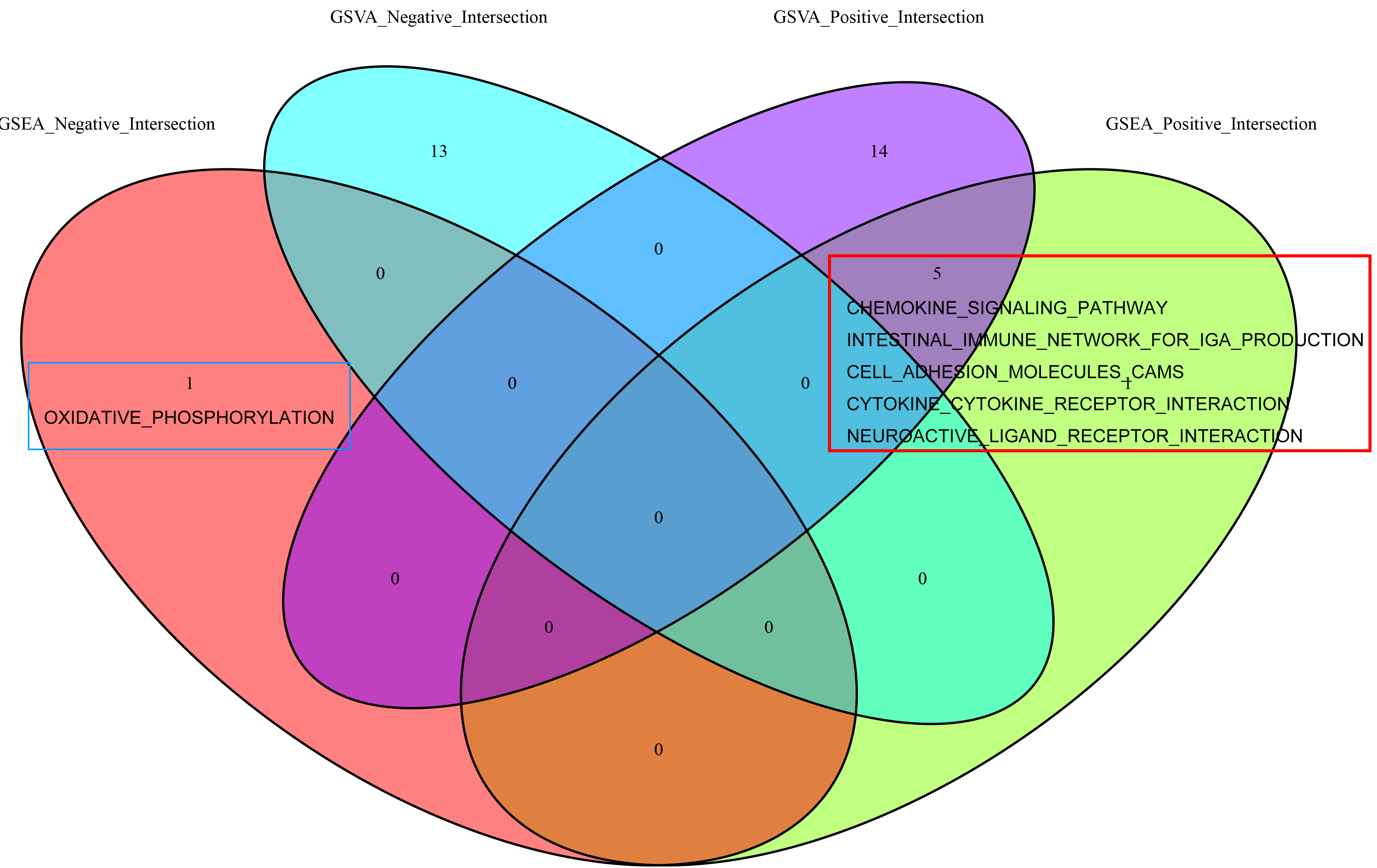

Supplement: Supplementary file 1 [file biomolecules-14-00179-s001.zip › Supplementary Figure S11.jpg]

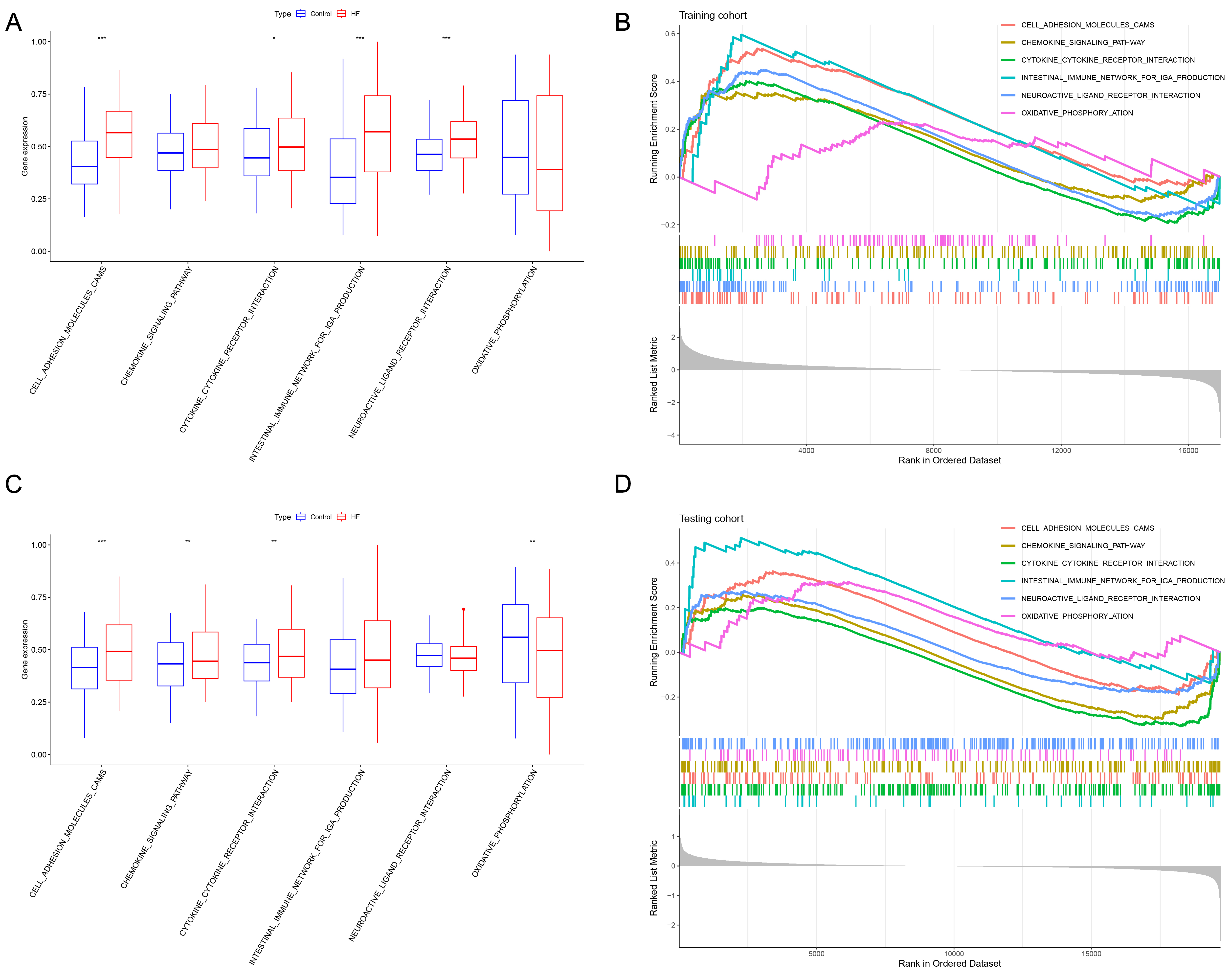

Supplement: Supplementary file 1 [file biomolecules-14-00179-s001.zip › Supplementary Figure S12.jpg]

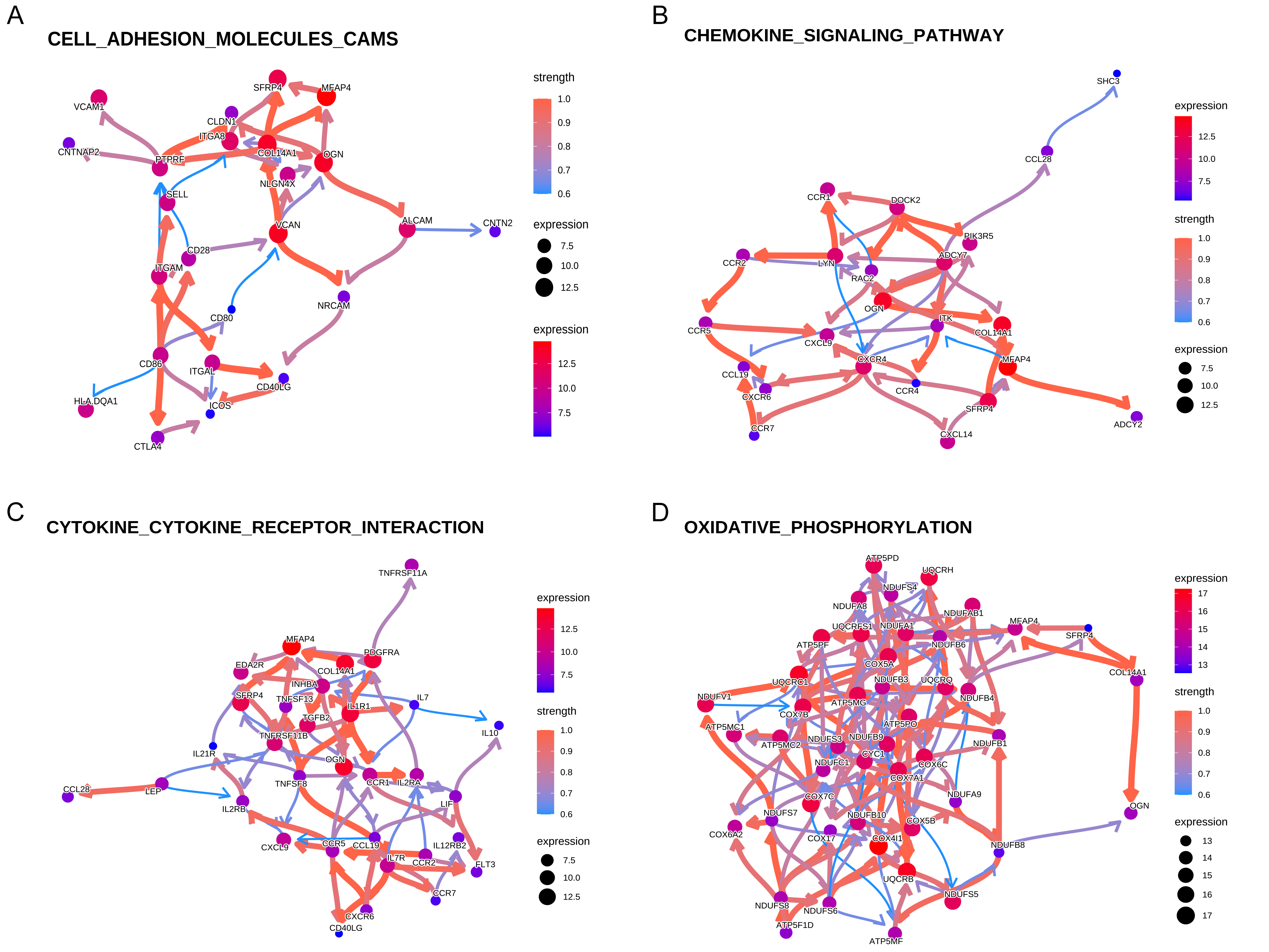

Supplement: Supplementary file 1 [file biomolecules-14-00179-s001.zip › Supplementary Figure S13.jpg]

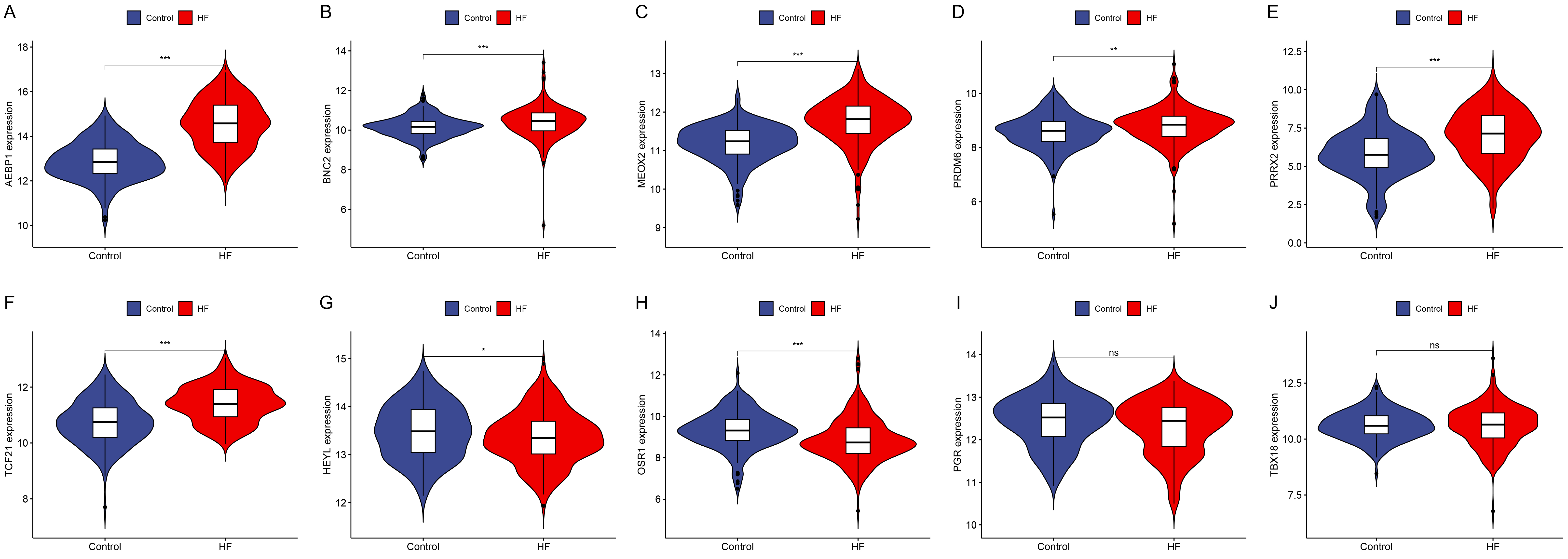

Supplement: Supplementary file 1 [file biomolecules-14-00179-s001.zip › Supplementary Figure S14.jpg]

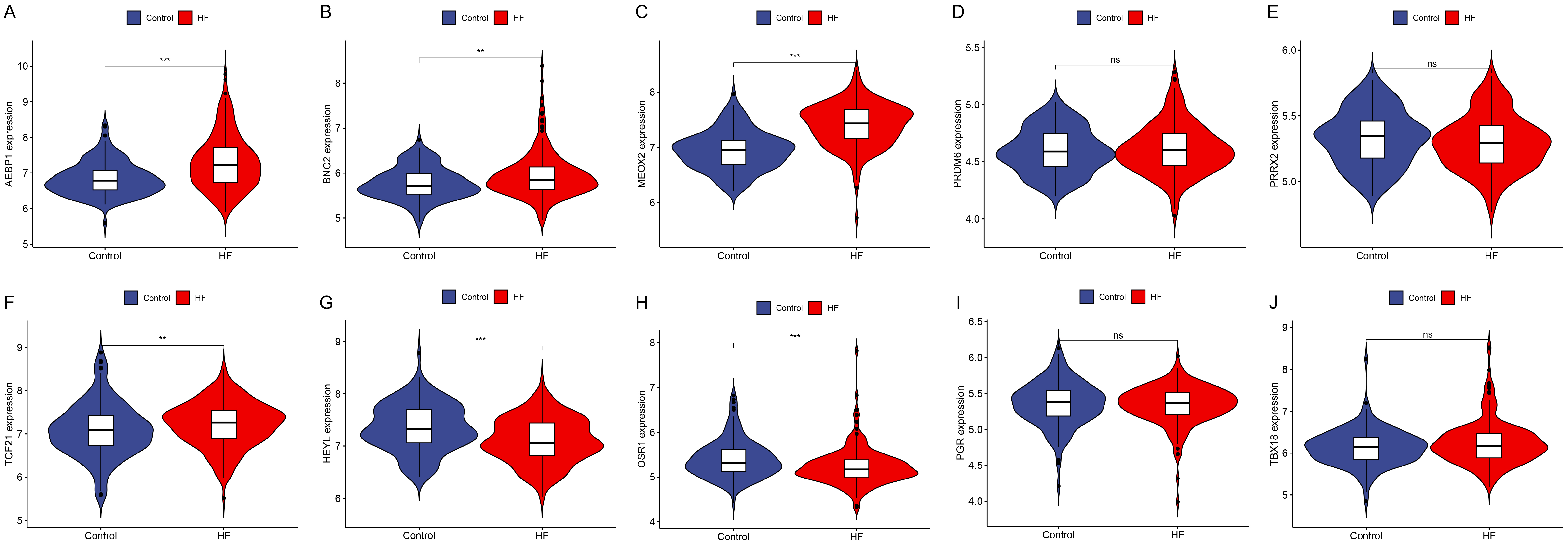

Supplement: Supplementary file 1 [file biomolecules-14-00179-s001.zip › Supplementary Figure S15.jpg]

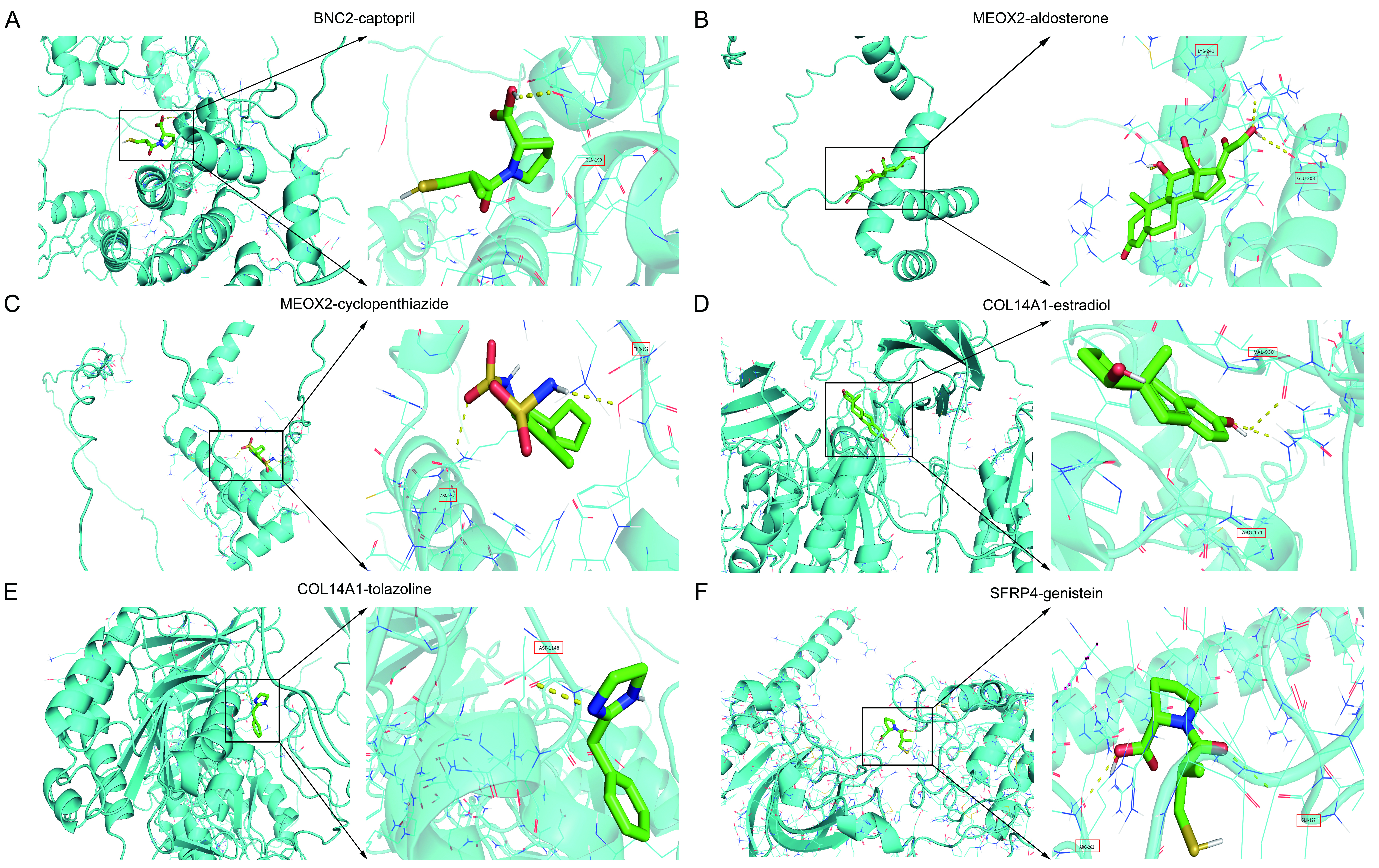

Supplement: Supplementary file 1 [file biomolecules-14-00179-s001.zip › Supplementary Figure S16.jpg]

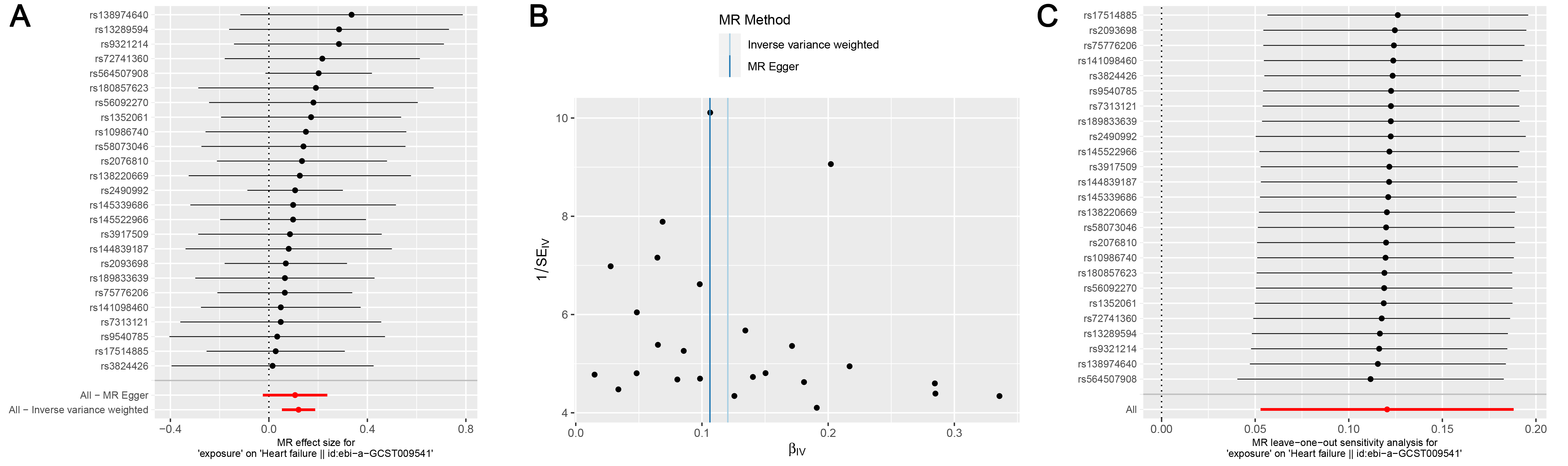

Supplement: Supplementary file 1 [file biomolecules-14-00179-s001.zip › Supplementary Figure S17.jpg]

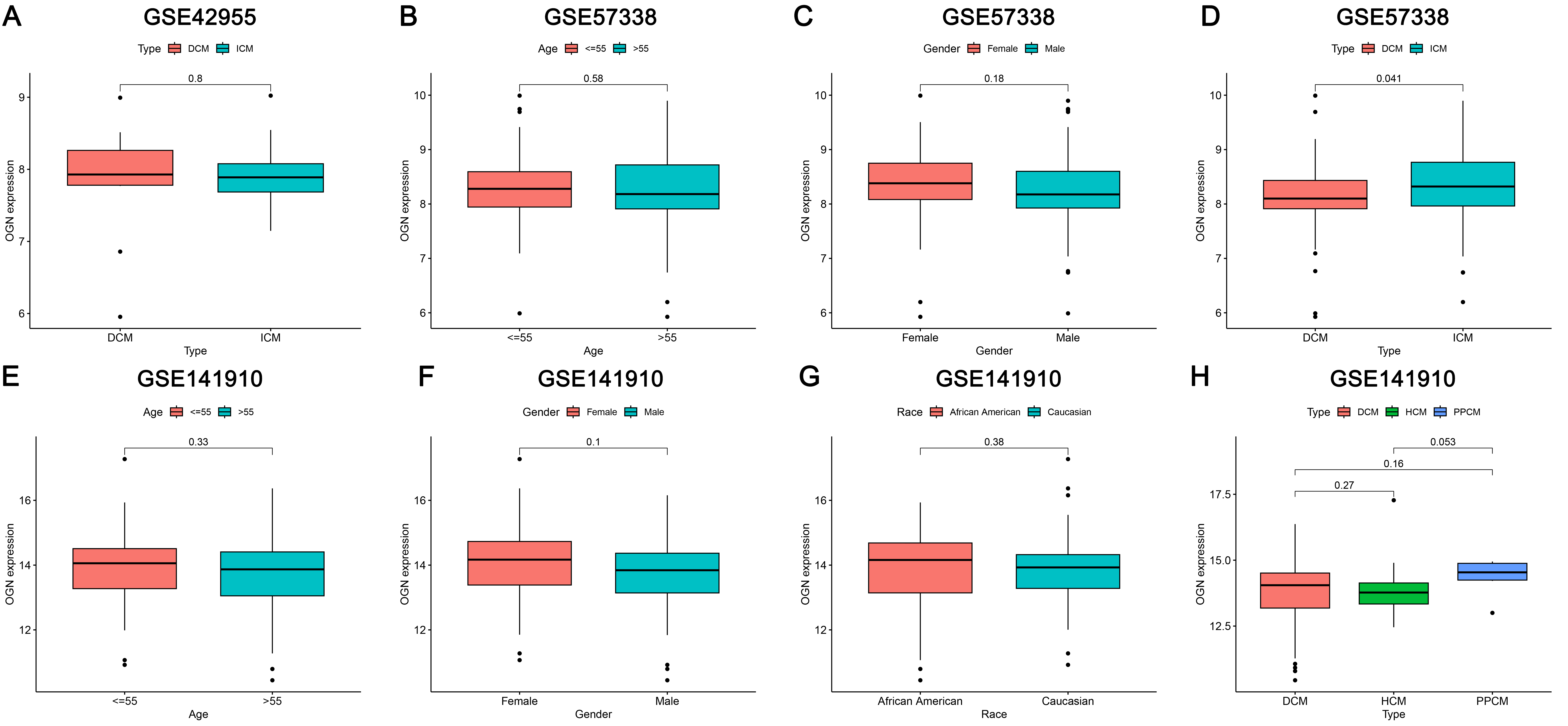

Supplement: Supplementary file 1 [file biomolecules-14-00179-s001.zip › Supplementary Figure S18.jpg]

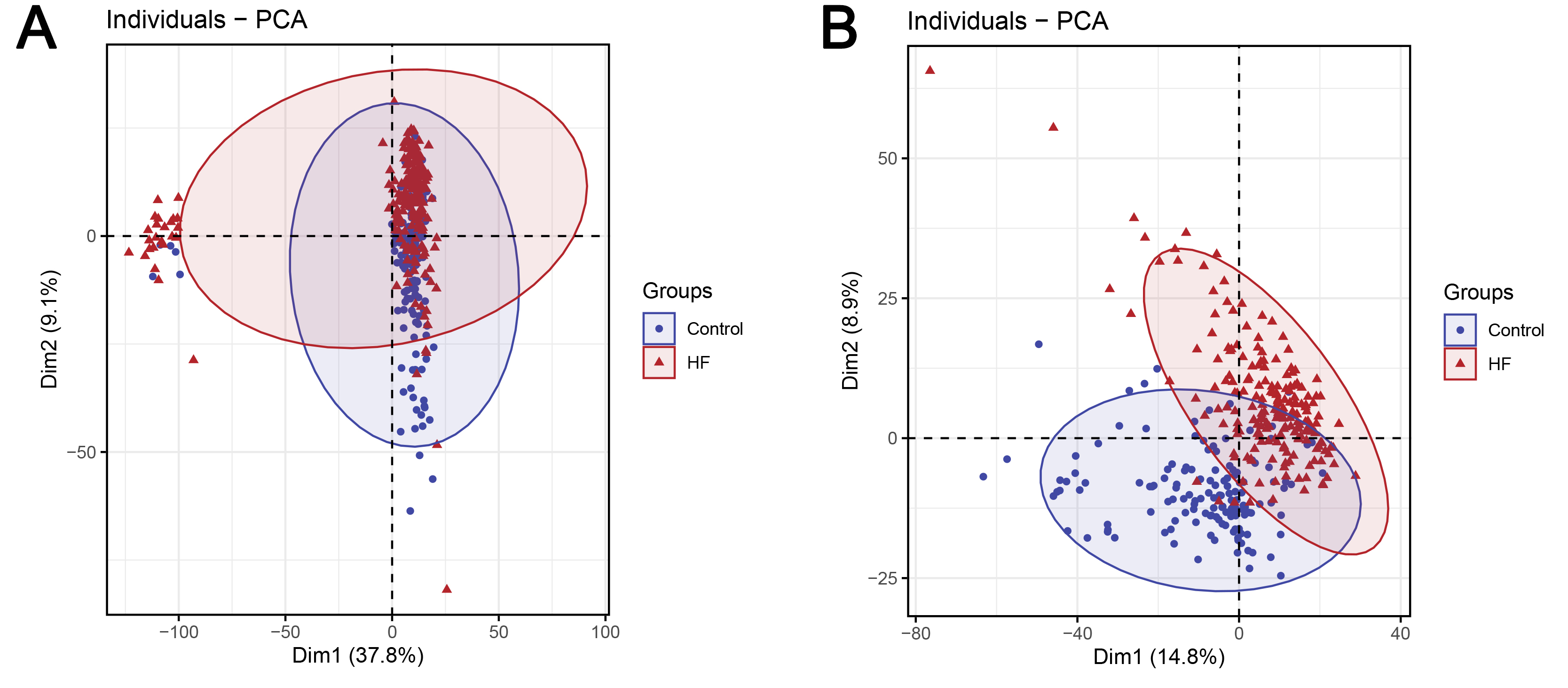

Supplement: Supplementary file 1 [file biomolecules-14-00179-s001.zip › Supplementary Figure S2.jpg]

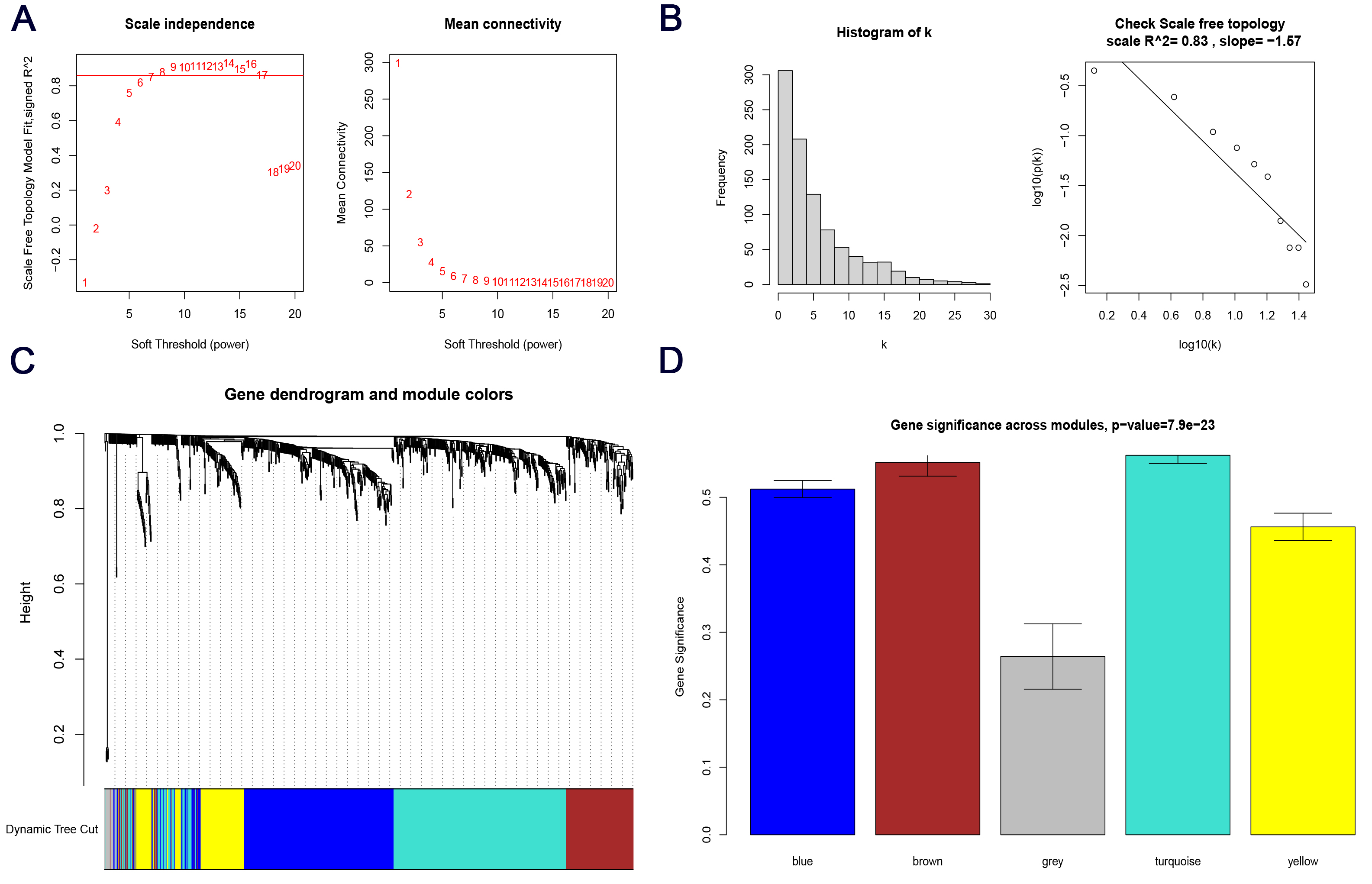

Supplement: Supplementary file 1 [file biomolecules-14-00179-s001.zip › Supplementary Figure S3.jpg]

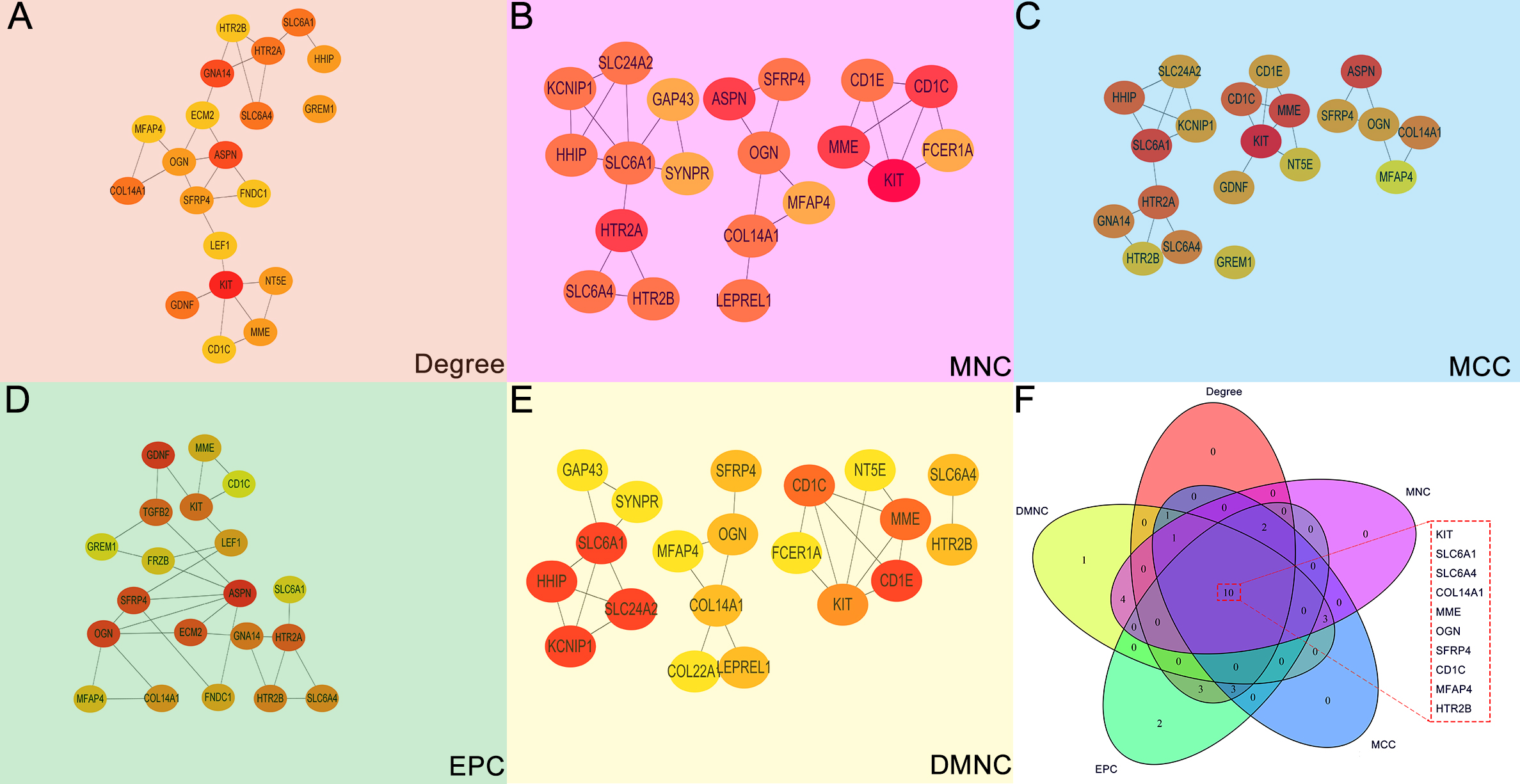

Supplement: Supplementary file 1 [file biomolecules-14-00179-s001.zip › Supplementary Figure S4.jpg]

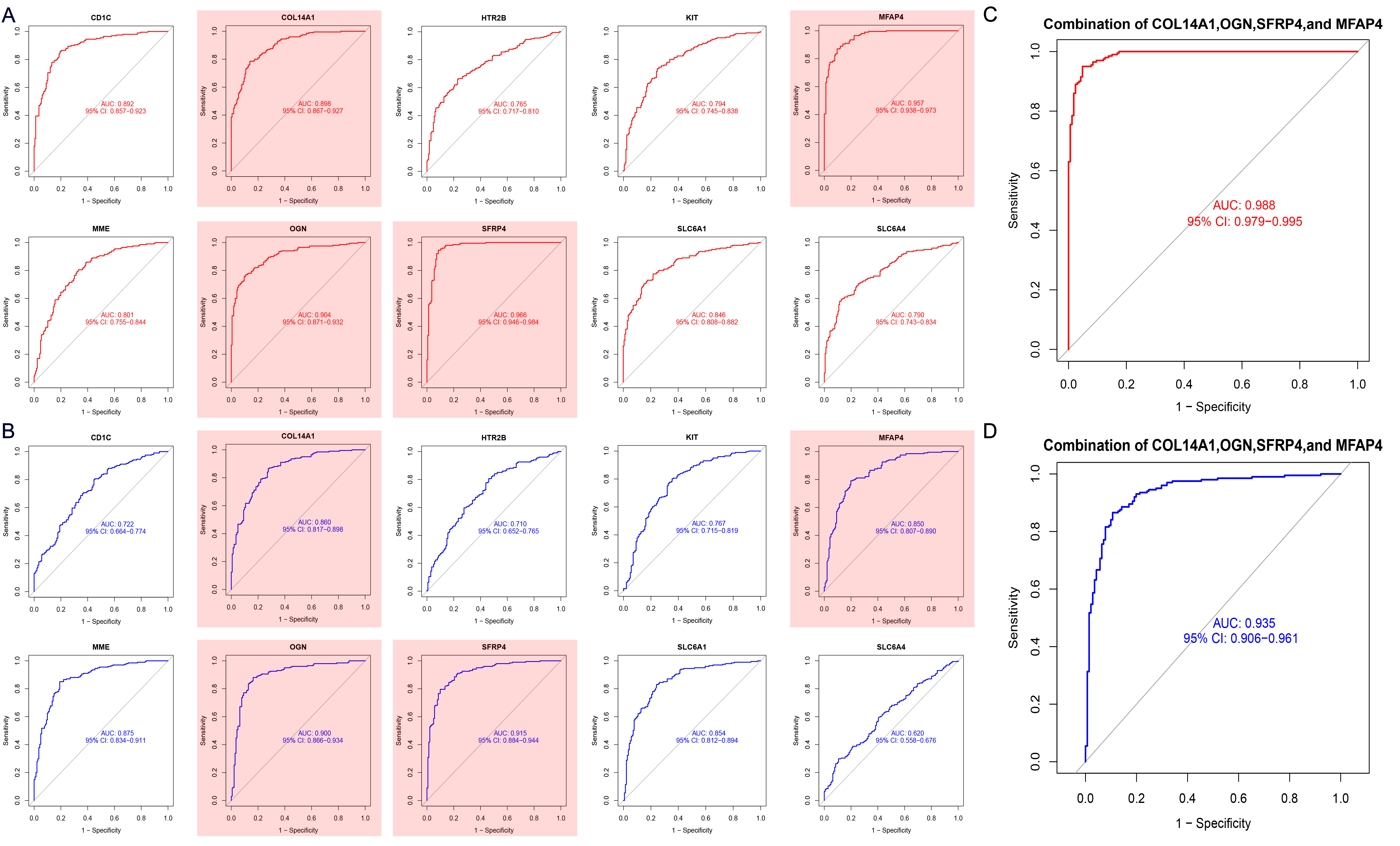

Supplement: Supplementary file 1 [file biomolecules-14-00179-s001.zip › Supplementary Figure S5.jpg]

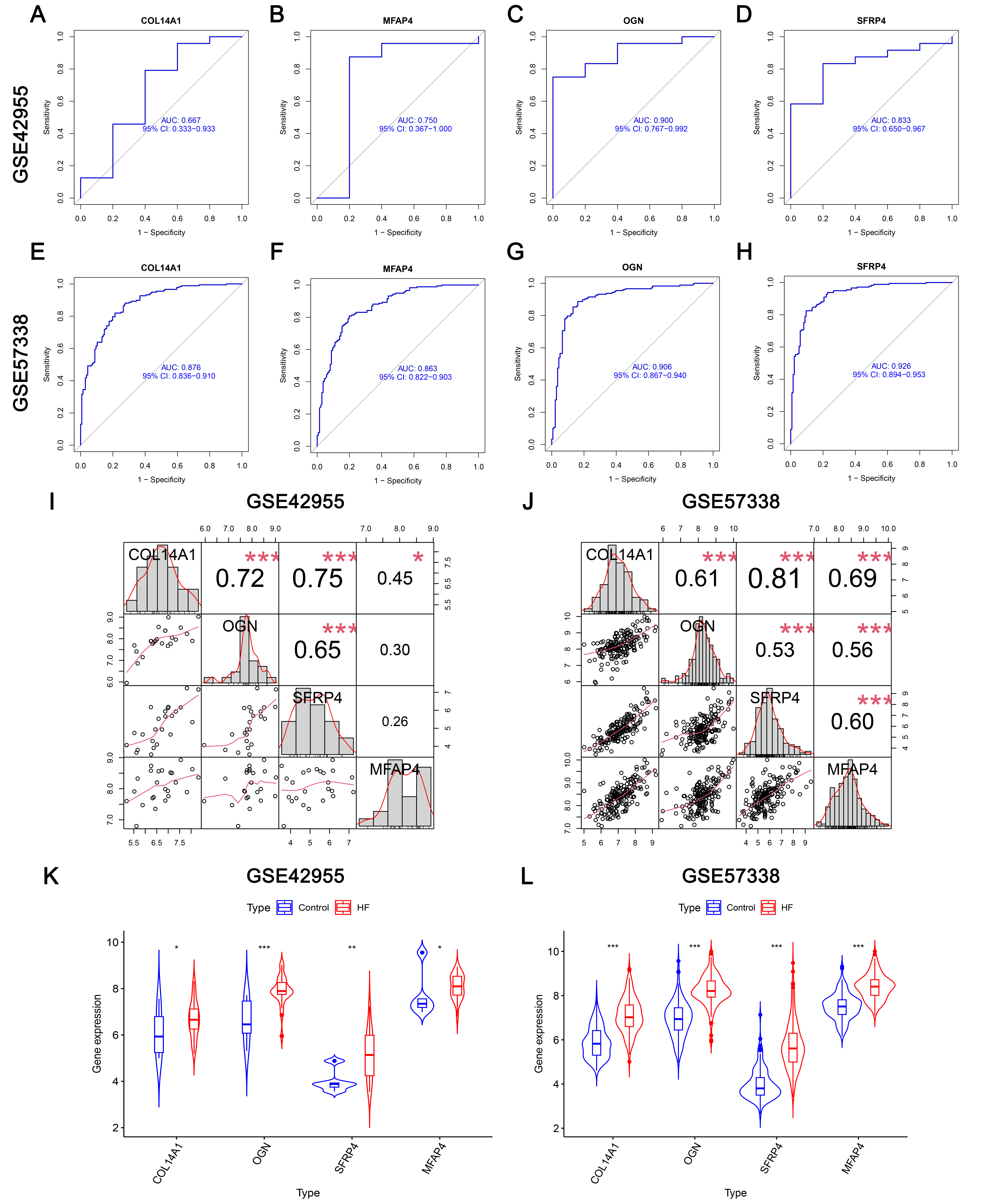

Supplement: Supplementary file 1 [file biomolecules-14-00179-s001.zip › Supplementary Figure S6.jpg]

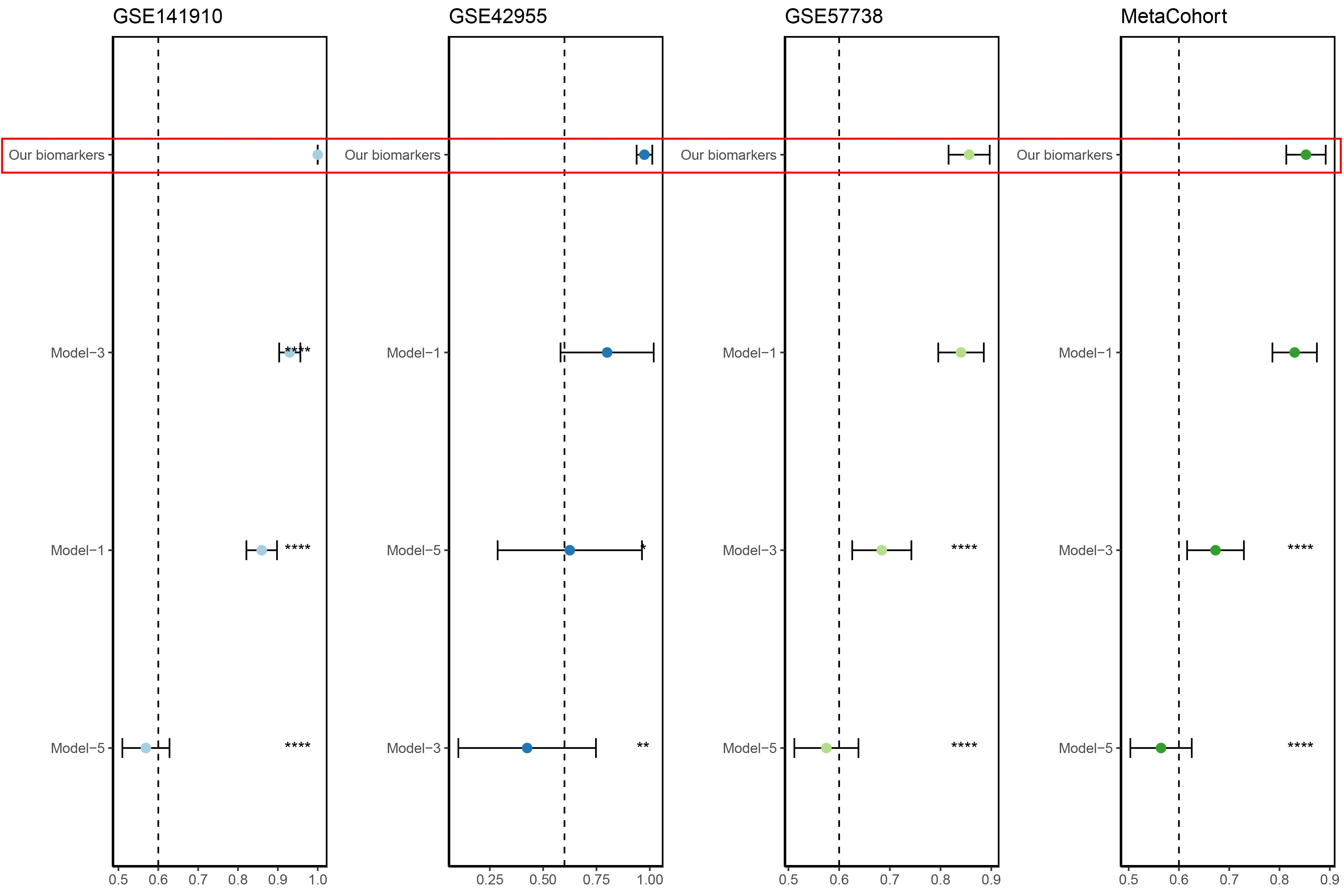

Supplement: Supplementary file 1 [file biomolecules-14-00179-s001.zip › Supplementary Figure S7.jpg]

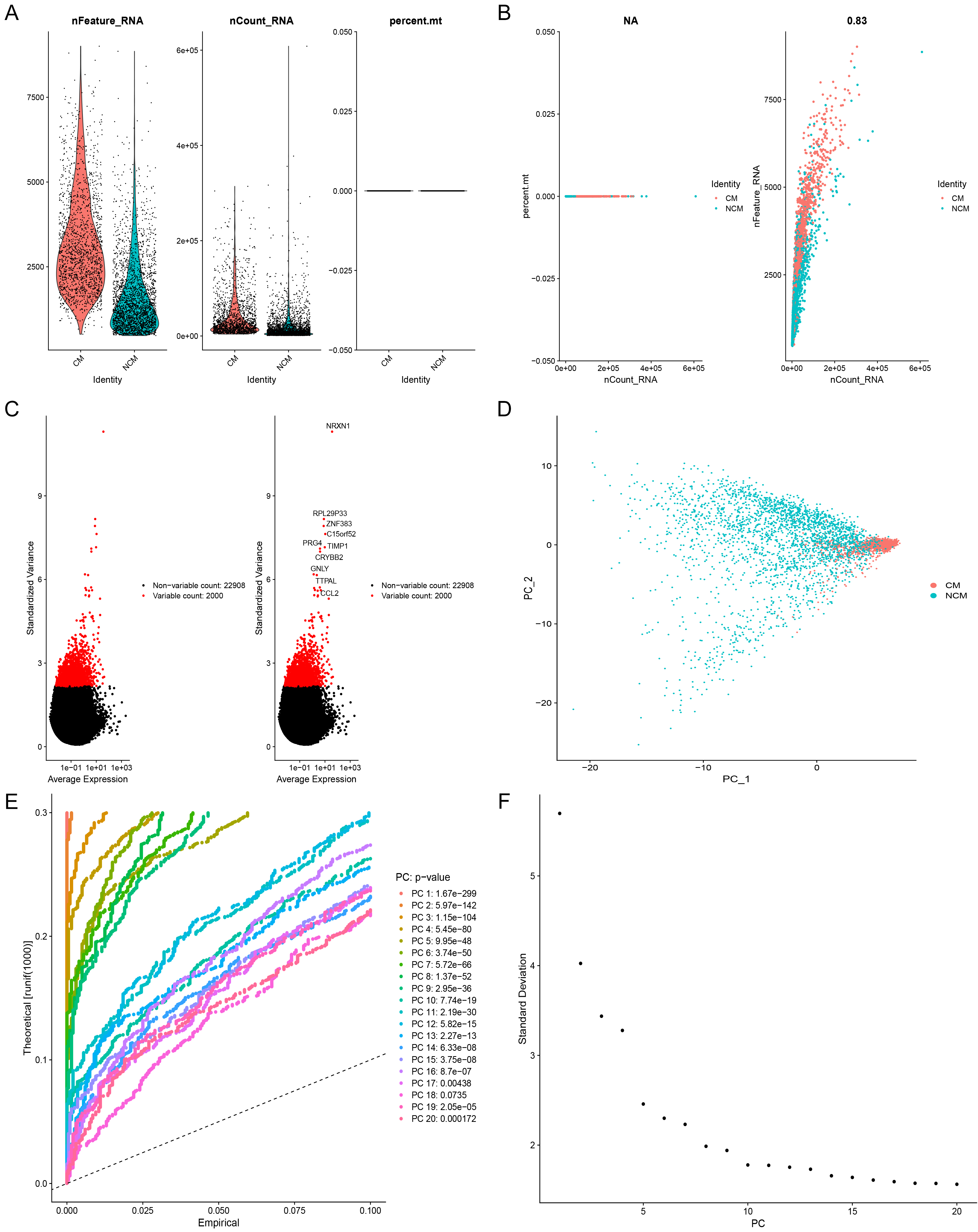

Supplement: Supplementary file 1 [file biomolecules-14-00179-s001.zip › Supplementary Figure S8.jpg]

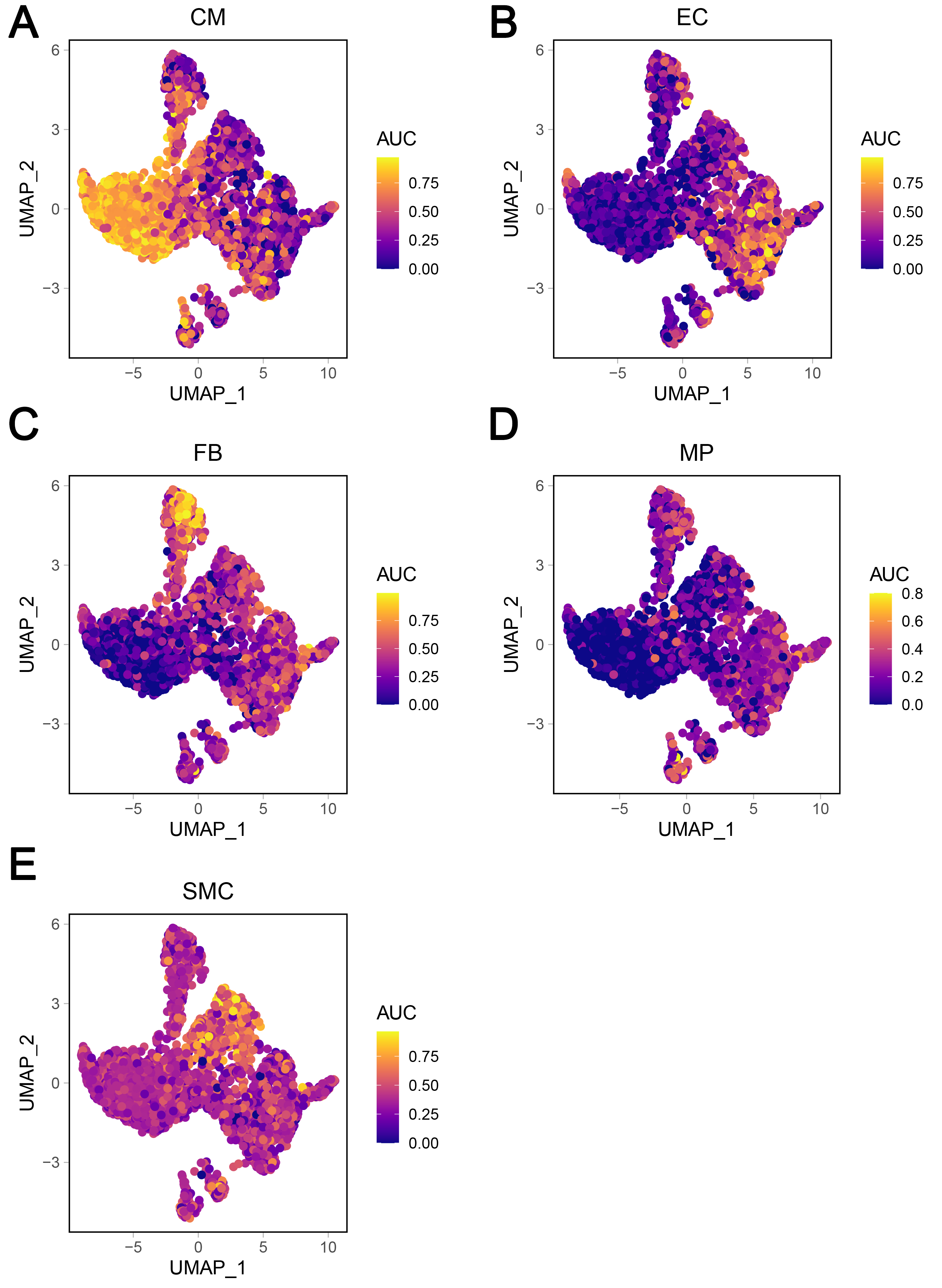

Supplement: Supplementary file 1 [file biomolecules-14-00179-s001.zip › Supplementary Figure S9.jpg]
